# Ecological niches in the polyploid complex *Linum suffruticosum s.l.*

**DOI:** 10.3389/fpls.2023.1148828

**Published:** 2023-04-19

**Authors:** Ana Afonso, Sílvia Castro, João Loureiro, Juan Arroyo, Albano Figueiredo, Sara Lopes, Mariana Castro

**Affiliations:** ^1^ Centre for Functional Ecology, Department of Life Sciences, University of Coimbra, Coimbra, Portugal; ^2^ Departamento de Biología Vegetal y Ecología, Universidad de Sevilla, Sevilla, Spain; ^3^ Centre of Studies in Geography and Spatial Planning (CEGOT), Department of Geography and Tourism, University of Coimbra, Coimbra, Portugal

**Keywords:** ecological niche, linum, Mediterranean region, niche modelling, polyploids

## Abstract

**Introduction:**

The high frequency of polyploidy in the evolutionary history of many plant groups occurring in the Mediterranean region is likely a consequence of its dynamic paleogeographic and climatic history. Polyploids frequently have distinct characteristics that allow them to overcome the minority cytotype exclusion. Such traits may enable polyploid individuals to grow in habitats different from their parentals and/or expand to new areas, leading to spatial segregation. Therefore, the successful establishment of polyploid lineages has long been associated with niche divergence or niche partitioning and the ability of polyploids to cope with different, often more stressful, conditions. In this study, we aimed to explore the role of environmental variables associated with the current distribution patterns of cytotypes within the polyploid complex *Linum suffruticosum s.l.*.

**Methods:**

The distribution and environmental niches of the five main cytotypes of *Linum suffruticosum s.l.* (diploids, tetraploids, hexaploids, octoploids and decaploids) were studied across its distribution range. Realized environmental niche of each cytotype was determined using niche modelling tools, such as maximum entropy modelling and niche equivalency and similarity tests.

**Results:**

Differences in the environmental conditions of *L. suffruticosum s.l.* cytotypes were observed, with polyploids being associated with habitats of increased drought and soil pH, narrower temperature ranges and decreased soil water and cation exchange capacities. Diploids present the widest environmental niche, and polyploids occupy part of the diploid niche. Although some polyploids have equivalent potential ecological niches, cytotypes do not co-occur in nature. Additionally, the ecological niche of this polyploid complex is different between continents, with North African habitats being characterised by differences in soil texture, higher pH, and low cation exchange capacity, precipitation and soil water capacity and higher temperatures than habitats in southwest Europe.

**Discussion:**

The different ecological conditions played a role in the distribution of cytotypes, but the mosaic distribution could not be entirely explained by the environmental variables included in this study. Other factors, such as reproductive isolation and competitive interactions among cytotypes, could further explain the current diversity and distribution patterns in white flax. This study provides relevant data on the niche requirements of each cytotype for further competition and reciprocal transplant experiments. further competition and reciprocal transplant experiments.

## Introduction

Polyploidization is a widespread mechanism of plant evolution and diversification (Soltis and Soltis, 1999; Soltis et al., 2010; Castro and Loureiro, 2014). Whole genome duplications (WGD) have occurred multiple times during the evolutionary history of angiosperms (Grant, 1981; Soltis, 2005; Van De Peer et al., 2017; Landis et al., 2018; Porturas et al., 2019), with studies suggesting that 47% to 100% underwent a WGD event during its evolutionary history (Grant, 1981; Masterson, 1994; Cui et al., 2006; Soltis et al., 2009). Due to its broad-scale consequences on gene expression and developmental processes, WGDs are known to lead to remarkable shifts in genetic, phenotypic and/or physiological traits that can confer advantages to the newly formed polyploids (Levin, 2002; Husband et al., 2013; Barker et al., 2016; Porturas et al., 2019; Van De Peer et al., 2020).

The spatial distribution of cytotypes results from several, often complex, interacting processes occurring in natural populations, such as cytotype origin, formation rates, ecological preferences, vegetative propagation and apomixies, competitive and dispersal abilities, and inter-cytotype reproductive interactions (Levin, 2002). In nature, for a polyploid to establish, it must have distinct reproductive and competitive characteristics that allow the polyploid to overcome the numerical disadvantage within the progenitor’s population (minority cytotype exclusion; Levin, 1975; Fowler and Levin, 1984; Husband, 2000; Levin, 2002). In many polyploid complexes, differences in traits have enabled polyploid individuals to grow and/or colonise habitats different from their parentals, leading to spatial segregation (Balao et al., 2009; Kolář et al., 2009; Glennon et al., 2012). Among the traits that might have played a significant role in spatial segregation is the ability of polyploids to cope with more stressful conditions. For example, higher tolerance to low nutrient levels, drought, and cold temperatures has been proposed in several studies (Levin, 2002; Maherali et al., 2009; Hao et al., 2013; Thompson et al., 2014). Therefore, the successful establishment of polyploid lineages has long been associated with niche divergence or niche partitioning (Levin, 1975; Glennon et al., 2014; Thompson et al., 2014; Muñoz-Pajares et al., 2018).

The Mediterranean Basin is known for its complex geological and paleoclimatic history. It is an extensive territory around the Mediterranean Sea characterised by a Mediterranean climate, *i.e.*, with mild, rainy winters and hot, dry summers (Thompson, 2020). The Mediterranean region is considered a biodiversity hotspot (Zachos and Habel, 2011), with estimates of polyploidy incidence of 36.5%, with higher values being detected for the Iberian Peninsula (48.8%; Marques et al., 2018). The high frequency of polyploids in the evolutionary history of many plants groups from this region is likely a consequence of its dynamic paleogeographic and climatic history (*e.g.*, Late Miocene Salinity Crisis, the spread of Mediterranean-type climate at the Pliocene, Pleistocene Ice Ages) (Thompson, 2020), and ecogeographical heterogeneity (Blondel et al., 2010). In the Iberian Peninsula, the determinant factors of the evolution of plant lineages and polyploid complexes include the existence of mountain ranges that promoted multiple refugia and produced allopatric and parapatric clades and the recurrent connections and disconnections with Northern Africa (Hewitt, 2011; Nieto-Feliner, 2014; Thompson, 2020).

The development of niche modelling tools, such as ecological niche modelling (ENM; Warren et al., 2008) and multivariate analyses of niche variables (Broennimann et al., 2012), enables to explore the environmental preferences of different cytotypes and to study the patterns of spatial segregation. These tools are based on a quantitative assessment of ecological divergence related to geographic distribution and statistical comparison of the overlap of the niche occupied by different cytogenetic entities. Therefore it allows for building hypotheses for the mechanisms involved in cytotype establishment and subsequent spread (Warren et al., 2008; Broennimann et al., 2012). Many studies have characterised the abiotic factors of polyploidy populations and evaluated cytotype environmental preferences, predicting the possible existence of niche shifts (Thompson et al., 2014; Visger et al., 2016; Muñoz-Pajares et al., 2018; López-Jurado et al., 2019; Castro et al., 2020) or niche conservatism (McIntyre, 2012; Laport et al., 2013; Glennon et al., 2014; Castro et al., 2019), between polyploids and their progenitors. For example, in *Chamerion angustifolium* L. Holub, it was shown that tetraploids occupied warmer and drier habitats than diploid progenitors (Thompson et al., 2014), while in *Erysimum mediohispanicum* Polatschek tetraploids grow in habitats with higher levels of precipitation than diploids (Muñoz-Pajares et al., 2018). However, other studies showed no niche differentiation, suggesting that other factors such as competitive and dispersal abilities and inter-cytotype reproductive barriers were involved in the success of polyploids (Levin, 2002; Godsoe et al., 2013; Laport et al., 2013; Kolář et al., 2017; Castro et al., 2018; Morgan et al., 2020).

**Figure 1:**
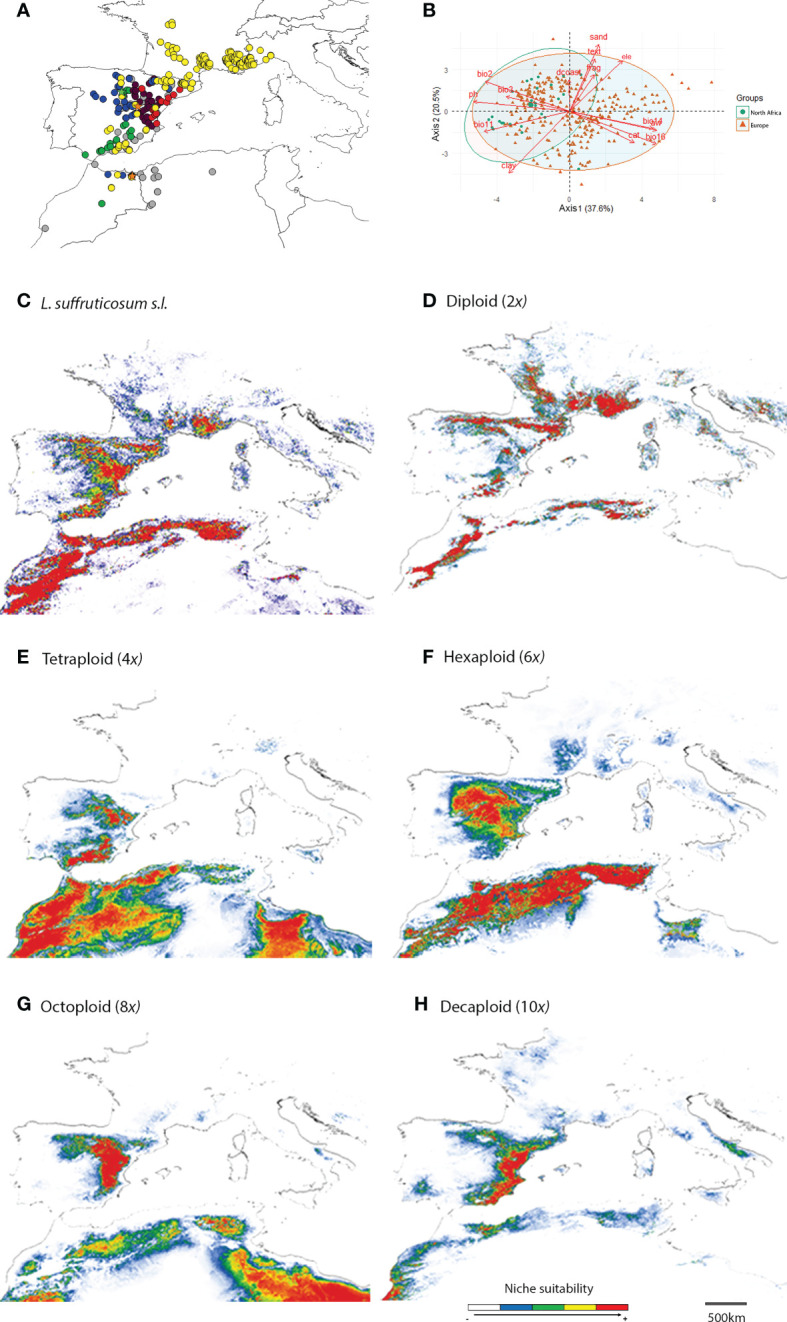
Distribution of *Linum suffruticosum s.l.* cytotypes: diploids, yellow circles; tetraploids, green circles; hexaploids, blue circles; octoploids, purple circles; decaploids, red circles; diploid-tetraploid mixed-ploidy population, orange star; hexaploid-tetraploid mixed-ploidy population, green star; without cytotype information, grey circles **(A)**; Principal Component Analysis (PCA) of all variables (Precipitation of Driest Month, bio14; Precipitation of Wettest Quarter, bio16; Mean Temperature of Coldest Quarter, bio11; Mean Diurnal Range, bio2; Isothermality, bio3; elevation, ele; Distance to the coast, dcoast; soil water capacity at 15 cm, aw; Fragment content at 15 cm, frag; Clay content at 15 cm, clay; Soil pH at 30 cm, ph; Sand content at 15 cm, sand; Cation exchange capacity at 15 cm, cat; Soil texture at 15 cm, text) for Europe, and North Africa **(B)**; and habitat suitability for *L. suffruticosum s.l.* **(C)**, and for diploids **(D)**; tetraploids **(E)**; hexaploids **(F)**; octoploids **(G)**, and decaploids **(H)** separately.

*Linum suffruticosum s.l.* is a polyploid complex distributed through the western Mediterranean basin (Rogers, 1979; Nicholls, 1985a; Nicholls, 1985b; Nicholls, 1986; McDill et al., 2009). Recent detailed studies have shown that *L. suffruticosum s.l.* harbors a high cytogenetic diversity, with five major cytotypes [namely diploids (2*x*), tetraploids (4*x*), hexaploids (6*x*), octoploids (8*x*) and decaploids (10*x*)] being detected in nature (Afonso et al., 2021). The different ploidy levels are distributed parapatrically, geographically structured, and comprise several contact zones with very few mixed-ploidy populations (15.0%, i.e., 23 out of 151 populations, **Figure 1A**), usually with a dominant cytotype (Afonso et al., 2021). The cytogenetic diversity was found in the Iberian Peninsula and North Africa, with the remaining areas of the species distribution in Europe (S France and NW Italy) being characterised by homogeneously diploid populations only (Afonso et al., 2021). *L. suffruticosum s.l.* has a high cytogenetic diversity with a complex mosaic distribution distributed along the W Mediterranean basin and constitutes an ideal system to explore the role of niche divergence in explaining the current distribution patterns of different cytotypes.

The main objective of this study was to explore the role of environmental variables in the current distribution of cytotypes within the polyploid complex *L. suffruticosum s.l*. Given the group’s cytogenetic variability and wide distribution range, and the potential impact of WGDs in physiological traits and environmental tolerances, we hypothesised that polyploidisation may have led to shifts in environmental preferences. Thus, diploids and polyploids are expected to colonise different environmental niches, resulting in a low geographic overlap. By coupling information about cytotype diversity, geographical patterns, and environmental preferences, this study will formulate ecologically-driven hypotheses that might help explaining the establishment and spread of *L. suffruticosum s.l.* cytotypes and polyploid lineages in general.

## Materials and methods

### Study system

*Linum suffruticosum s.l.* occurs mostly on limestone soils, in Mediterranean climate areas in the mountains to lowlands and dry regions. The geographic distribution comprises the western Mediterranean basin, from the Iberian Peninsula to the northwestern Italy, and northwestern Africa, where it is less abundant (A. Afonso, field observations). The group comprises perennial plants and presents a complex reproductive strategy. Populations are heterostylous, comprising both long- and short-styled morphs and sexual reproduction is obligatory due to a heteromorphic self-incompatibility system associated with the style-length dimorphism (Rogers, 1979; Armbruster et al., 2006; Afonso, 2022). The reciprocity of sex organs in *L. suffruticosum s.l.* has been described to be three-dimentional as a result of differences in the angle of divergence of styles and stamens from the flower central axis and of the degree of rotation of styles and filaments. The stigmas of short-styled morph contact the ventral side of the pollinator and the stigmas of long-styled morph contact the dorsal side. By opposition, the pollen from the short-styled morph is placed in the dorsal side of the pollinator, while the pollen from long-styled morph is placed in the ventral side (Armbruster et al., 2006). Additionally, vegetative propagation is almost negligible.

*Linum suffruticosum s.l.* is a diploid-polyploid complex with high cytogenetic and morphological variability (*e.g.*, Rogers et al., 1972; Elena Roselló et al., 1985; Nicholls, 1986; Afonso et al., 2021). Furthermore, this group revealed a complex taxonomy due to its high morphological variability and lack of reliable diagnostic characters (López-Gonzalez, 1979; Martínez-Labarga and Garmendia, 2015). The lack diagnostic characters and the high diversity have led to different taxonomic treatments over the years. The most recent treatment recognized more than 20 taxa for the Iberian Peninsula alone (Martínez-Labarga and Garmendia, 2015), while in most of the previous treatments, only three taxa have been consensually accepted as distinct species (*L. salsoloides* Lam., *L. appressum* Caball. and *L. suffruticosum*), with some varieties being described in *L. suffruticosum* (Jahandiez and Maire, 1932; Ockendon and Walters, 1968; López-Gonzalez, 1979). The identification made here is based on Afonso et al. (2021) that identified the populations of *L. suffruticosum s.l.* according to López-Gonzalez (1979) and Fennane et al. (2007) and assigned the speciemns into four taxa: *L. suffruticosum* var. *milletii* (Sennen & Gonzalo) G.López, *L. suffruticosum s.s.*, *L. salsoloides* and *L. appressum-salsoloides* (including plants that could not be clearly assigned to either of these two species). Besides the difficulties in identifying some specimens as *L. appressum* or *L. salsoloides*, Afonso et al. (2021) also identified intermediate population with morphological characters between *L. suffruticosum*, *L. salsoloides* and *L. appressum*. Populations from Morocco were classified as *L. suffruticosum* following the available literature for this region (Jahandiez and Maire, 1932; Emberger and Maire, 1941; Quézel and Santa, 1962; Fennane et al., 2007; Valdés et al., 2007).

The chromosome base number can be n = *x* = 8 or n = *x* = 9, with the latter being the most common. Diploid populations cover a larger area, being detected throughout all sampled areas, and it is the only cytotype found north and northeast of the Pyrenees. Most cytogenetic diversity is found in the Iberian Peninsula and North Africa, with tetraploids, hexaploids, octoploids, and decaploids being detected (Afonso et al., 2021). Additionally, recent studies of pollen tube development after controlled hand pollinations support the maintenance of self- and morph-icnompatibility system in *L. suffruticosum* polyploids (Afonso, 2022).

### Ocurrence data

For this study, occurrence records of *L. suffruticosum s.l.* populations and their ploidy level were mostly based in Afonso et al. (2021). Several new populations were sampled, with field sampling and ploidy level of the sampled individuals being estimated (when possible) using flow cytometry following Afonso et al. (2021). Further occurrences for North Africa were obtained from the GBIF database (http://gbif.org). In total, 324 natural field collected populations were selected: 64 diploid (4 from North Africa and 60 from Europe), 26 tetraploid (5 from North Africa and 21 from Europe), 24 hexaploid (2 from North Africa and 22 from Europe), 25 octoploid and 14 decaploid populations from Europe, 2 diploid-tetraploid mixed-ploidy populations (1 from North Africa and 1 from Europe) 1 tetraploid-hexaploid population from Europe. These populations were obtained from Afonso et al., 2021, where genome size and DNA ploidy were assessed using flow cytometry. In addition, 73 diploid populations with genome size and DNA ploidy estimates using flow cytometry and 95 populations (5 from North Africa and 90 from Europe) without known ploidy level were also added to this study. Finally, 13 occurrences data from GBIF (all observational occurrences) were also used (Appendix 1, **Figure 1A** and Afonso et al., 2021).

### Environmental data

This study used a Grinnellian niche concept, with only abiotic variables considered to define each cytotype niche. Given that the whole spectrum of environmental and community conditions was not analysed, a realised niche concept for the cytotypes was assumed, which resulted from interactions with other species and cytotypes (Soberón, 2007).

To explore the environmental niches of *L. suffruticosum s.l.* cytotypes, 19 bioclimatic variables from the WorldClim database (https://www.worldclim.org), and 18 topographic and soil conditions variables at two different depths (15 and 30 cm) from the World Soil Information (https://www.isric.or) were extracted at a resolution of 30 arc-seconds (approx. 1 km) for most of the distribution area of *L. suffruticosum s.l.* (27.0° to 51.0 N latitude, -13.0° to 18.0° longitude). To evaluate the contribution of each variable to the total reported variance, exploratory Principal Component Analyses (PCA) were done, and correlations between the variables were obtained using Pearson or Spearman coefficients (for variables with continuous measurements or with ordinal scale, respectively; Appendix 2). Only one variable was selected for the pairs of variables with correlation values higher than 0.7. Therefore, the following non-correlated variables were used in niche modelling analyses: mean diurnal range (bio2); isothermality (bio3), mean temperature of the coldest quarter (bio11), precipitation of the driest month (bio14), precipitation of the wettest quarter (bio16), elevation (ele), distance to the coast (dcoast); furthermore, seven soil variables at two standard depths predicted using the global compilation of soil ground observations (accuracy assessment of the maps is available in Hengl et al., 2017) were used: soil water capacity at 15 cm in volumetric fraction (aw), clay content at 15 cm in mass fraction (clay), cation exchange capacity at 15 cm (cat), fragment content at 15 cm in volumetric fraction (frag), sand content at 15 cm in mass fraction (sand), soil pH at 30 cm (ph), and soil texture at 15 cm (text- texture class in USDA system - https://www.nrcs.usda.gov). In addition, soil texture was used as a categoric variable.

Values for climatic, topographic and soil variables were extracted for all the *L. suffruticosum s.l.* populations using the R package *dismo* (Hijmans et al., 2017). Given the major geograohical barrier between the two continents, to explore different environmental pressures between continents (Europe vs North Africa), generalised linear models (GLMs) were used with the continent as a fixed factor and each variable as a response variable. Furthermore, to assess differences among the cytotype’s environmental variables, generalised linear models (GLMs) were used with the cytotype as a fixed factor and each variable as a response variable. A Gaussian distribution with an identity link function was used for continuous variables and a Poisson distribution with a log link function was used for discrete variables. Soil water capacity, clay content, fragment content and sand content are proportions and, thus, were transformed with the arcsine of the square root. Statistical analyses were performed in R software v.3.6.1 (R Core Development Team, 2019), using the packages *car* for Type-III analysis of variance (Fox et al., 2005), *glm* for generalised linear models (Hastie and Pregibon, 1992) and *multcomp* for multiple comparisons after Type- III analysis of variance (Hothorn et al., 2017).

### Niche modelling

Niche modelling tools were used to explore the ecological requirements of 1) *L. suffruticosum s.l.*, and 2) each of the five cytotypes of *L. suffruticosum s.l.* Niche modelling analyses were performed with maximum entropy modelling (MaxEnt; package *biomod2*; Thuiller et al., 2016). In both approaches, spatial predictive models were calibrated based on the selected variables and presence/absence data using European reports, as reports from North Africa were scarce for such a vast and heterogeneous area. Field and GBIF records were used to build the presence dataset. Duplicate occurrences were removed, and locally dense sampling was reduced by thinning the records to one per grid cell size. To obtain pseudo-absences (background points), we applied a buffer of 10 km around each reported population from the presence dataset, and 5000 points were randomly selected beyond this buffer; additionally, a filter of 1 km was used to remove pseudo-absences that were separated by less than this distance to avoid oversampling. The first approach used all European populations as presences and background points as absences. In contrast, in the second approach, populations of a given cytotype were recorded as presences and the populations of the other cytotypes and the background points were recorded as absences. Finally, mixed-ploidy populations were considered as presences for both cytotypes. Models were replicated 30 times after splitting data in training (70%) and testing (30%) subsets (Phillips et al., 2006; Araújo and New, 2007). To guarantee the statistical independence of all the replicates, each occurrence was used only once in each run, either as training or as a test occurrence (Phillips, 2008). Models were evaluated based on the independent accuracy measure of Area Under the Curve of the Receiver Operating Characteristic (AUC of ROC). Only models with AUC > 0.7 were considered for the final model. The evaluation of each model revealed high AUC values (mean ± SE; *L. suffruticosum s.l.*: 0.94 ± 0.01; 2*x*: 0.93 ± 0.01; 4*x*: 0.97 ± 0.04; 6*x*: 0.97 ± 0.02; 8*x*: 0.98 ± 0.02; 10*x*: 0.98 ± 0.02) and relatively low omission rates (mean ± SE; *L. suffruticosum s.l.*: 0.10 ± 0.04; 2*x*: 0.13 ± 0.05 and 4*x*: 0.04 ± 0.06; 6*x*: 0.01 ± 0.01; 8*x*: 0.01 ± 0.03; 10*x*: 0.00 ± 0.01), indicating that the models have high performance and capacity to distinguish suitable from unsuitable environmental conditions. In both approaches, and assuming that the environmental requirements of the species are similar over the Mediterranean basin, we used the final model and the 14 selected variables to project suitable areas of *L. suffruticosum s.l.* for North Africa and predict the total suitable habitat of the species and of each cytotype in that region. In the second approach, the final model of each cytotype was converted into a binary format (using the default threshold of 0.5), to calculate the suitable habitat of each cytotype and assess niche overlap (package *biomod2*; Thuiller et al., 2016).

### Niche equivalence and similarity tests

Niche equivalency and similarity tests (Warren et al., 2008; Broennimann et al., 2012), using Schoener’s *D* metric (Schoener, 1970), were applied to quantify niche overlap in the geographic distribution of cytotypes of *L. suffruticosum s.l.* in Europe. This metric ranges from 0 (no overlap) to 1 (complete overlap). The analyses were run with *ecospat* (Broennimann et al., 2012) and *raster* (Hijmans et al., 2017) packages using binary projections.

The *ecospat* R package was used to compare cytotype niches with an ordination approach using a PCA calibrated with environmental values (Di Cola et al., 2017). The PCA calculates the occurrence density and environmental factor density along environmental (principal component) axes for each pixel, maximising the ecological variance of cytotype areas. Then, the PCA scores of the two cytotype distributions being compared were projected onto a grid of cells bounded by the maximum and minimum PCA scores, which allowed the visual assessment of the overlap and dynamics of the environmental niches of cytotypes.

Both niche equivalency and similarity tests were computed for each pair of cytotypes to test whether predicted distributions were significantly different between cytotypes (classification by Warren et al., 2008; Smith and Donoghue, 2010; Broennimann et al., 2012). The niche identity test determines if the distribution models produced for the two cytotypes being compared differ in their environmental attributes by pooling records of two different cytotypes and by randomly sampling from the pooled occurrences to create a pseudo-replicate dataset of equal size that was then used for *D* calculation (simulated values). This process was repeated 100 times to obtain confidence intervals for evaluating the null hypothesis. For this, the simulated *D* values were compared with the observed *D* value, and the cytotype’s niches were considered equivalent if the observed *D* value fell within the 95^th^ percentile of the simulated *D* value (Broennimann et al., 2012). The niche similarity test determines whether the environmental niche of two different cytotypes are distinguishable by comparing the records of one cytotype with random points from the geographic range of the other cytotype. As in the identity test, the process was repeated 100 times to obtain confidence intervals.

All analyses were performed in R software version 3.0.1 (R Core Development Team, 2016). Quantum-GIS was used to observe and build the distribution maps.

## Results

### Ecological attributes of *L. suffruticosum s.l.*

*Linum suffruticosum s.l.* is found in habitats with highly variable ecological attributes (**Table 1**). It is located in a wide range of elevations (from 46 to 2599 m a.s.l.), precipitation ranges [bio 14 (1-69 mm) and bio16 (73-492 mm)] as well as close (2.09 km) and distant to the coast (379.36 km). For temperature variables, the range of values for isothermality (26-44) is more variable than the mean diurnal range (0.6-1.4 ˚C) and the mean temperature of the coldest quarter (-0.5-1.2 ˚C; **Table 1**). Regarding soil attributes, such as fragment, sand and clay content, soil pH, soil water capacity, soil texture and cation exchange capacity, this complex can be found in habitats with different conditions (**Table 1**). Some differences were found between European and North African habitats of *L. suffruticosum s.l.* in ecological attributes related to soil properties and climate. In North Africa, *L. suffruticosum s.l.* populations are found, on average, at a significantly higher elevation than in Europe, while in Europe, the plant occurs in a broader elevation range (**Table 1**). Statistically significant differences were found in precipitation and minimum temperatures, with rainfall being scarcer in North Africa and temperatures not reaching values as low as in Europe (**Table 1**). In North Africa, the soil pH is significantly more basic, and the soil water capacity is significantly lower than in Europe, while sand content values are considerably higher (**Table 1**). Regarding soil texture, there are predominantly different classes in Europe and North Africa (**Table 1**, texture class in USDA system - -https://www.nrcs.usda.gov). No statistically significant differences were observed for the remaining variables (**Table 1**).

**Table 1:** **Mean and standard error of the mean (mean ± SE) and minimum and maximum (min-max) values of selected variables used to characterise the niche of *Linum suffruticosum s.l.* in Europe and North Africa**.

Variables	CODE	*Linum suffruticosum s.l.*		Europe		North Africa		*F*_1,226_ value
		mean ± SE, N = 336	min-max	mean ± SE, N = 306	min-max	mean ± SE, N = 30	min-max	
Elevation (metres)	ele	896.5 ± 23.1	46.0-2599.0	880.5 ± 23.7	46.0-2599.0	1060.7 ± 91.6	269.0-2149.0	4.67*
Distance to the coast (km)	dcoast	131.3 ± 4.8	2.1-379.4	131.6 ± 4.9	2.1-379.4	128.3 ± 20.5	3.6-366.7	0.15 ^n.s^
Mean Diurnal Range (°C)	bio2	1.07 ± 0.01	0.60-1.40	1.06 ± 0.01	0.60-1.40	1.11 ± 0.03	0.60-1.30	1.49 ^n.s^
Isothermality (* 100)	bio3	38.3 ± 0.2	26.0-44.0	38.3 ± 0.1	28.0-44.0	38.3 ± 0.0	26.0-44.0	0.07 ^n.s^
Mean Temperature of Coldest Quarter (°C)	bio11	0.45 ± 0.01	-0.50-1.20	0.42 ± 0.01	-0.50-1.20	0.74 ± 0.05	0.20-1.20	54.07***
Precipitation of Driest Month (mm)	bio14	25.7 ± 1.0	1.0-69.0	27.8 ± 0.1	1.0-69.0	4.0 ± 0.4	1.0-9.0	60.83***
Precipitation of Wettest Quarter (mm)	bio16	219.3 ± 4.1	73.0-492.0	227.9 ± 4.1	73.0-492.0	131.7 ± 11.2	73.0-392.0	52.95***
Soil water capacity (v%)	aw	13.8 ± 0.1	10.0-18.0	14.0 ± 0.1	10.0-18.0	11.9 ± 0.2	10.0-14.0	38.13***
Cation exchange capacity (cmolc/kg)	cat	19.5 ± 0.2	13.0-30.0	19.6 ± 0.1	13.0-30.0	18.5 ± 0.6	14.0-24.0	3.67 ^n.s^
Soil pH (pH)	ph	7.0 ± 0.0	5.5-8.1	7.0 ± 0.0	5.5-8.1	7.6 ± 0.1	6.1-8.1	33.73***
Clay content (w%)	clay	24.51 ± 0.2	13.0-34.0	24.4 ± 0.2	13.0-34.0	25.6 ± 0.4	21.0-29.0	3.27 ^n.s^
Fragment content (v%)	frag	18.3 ± 0.2	8.0-28.0	18.2 ± 0.2	8.0-28.0	18.9 ± 0.7	8.0-24.0	0.79 ^n.s^
Sand content (w%)	sand	39.0 ± 0.3	23.0-59.0	38.7 ± 306.0	23.0-59.0	42.3 ± 0.7	36.0-51.0	12.30***
Soil texture (USDA system)	text	6.5 ± 0.1	4.0-9.0	6.5 ± 0.1	4.0-9.0	6.0 ± 0.3	4.0-7.0	3.95*

The number of populations (N), and F value and significance levels (n.s., P > 0.05, * P < 0.05, ** P < 0.01,*** P < 0.001) for the comparison between continents are also provided.

The two first PCA axes explained 58.1% (axis 1: 37.6%, axis 2: 20.5%) of the environmental variance in European and North African distribution. They revealed that the environmental values of North Africa overlapped with European environmental values, being the ecological attributes of European populations broader than those of North Africa. The latter coincided only partially with the cluster of European populations, being skewed along axis 1 due to higher values of pH, mean temperature of the coldest quarter (bio11), mean diurnal range (bio2) and clay content (clay), and lower levels of precipitation of the driest month (bio14), precipitation of wettest quarter (bio16), sand content (sand), soil texture (text), cation exchange capacity (cat) and soil water capacity (aw) (**Figure 1B**).

### Ecological attributes of *L. suffruticosum s.l.* cytotypes

When comparing environmental variables among cytotypes, significant differences were observed for all variables except for elevation, soil texture, clay and sand content (**Table 2** and Appendix 3). A gradient was observed for several variables, with increased ploidy associated with increasing mean diurnal temperature range, isothermality, mean temperature of coldest quarter, and soil pH, and decreasing precipitation values for the wettest quarter, soil water capacity, and cation exchange capacity (**Table 2**, Appendix 3).

**Table 2:** Mean and standard error of the mean (SE) of selected variables used to characterise the niche of *Linum suffruticosum s.l.* cytotypes.

Variables	Code	Diploids	Tetraploids	Hexaploids	Octoploids	Decaploids	F _4, 214_ value
		mean ± SE, N = 139	mean ± SE, N = 29	mean ± SE, N = 25	mean ± SE, N = 25	mean ± SE, N = 14	
Elevation (metres)	**ele**	866.9 ± 41.2	962.1 ± 66.9	796.6 ± 34.9	880.7 ± 54.4	601.0 ± 73.0	1.94^n.s.^
Distance to the coast (km)	**dcoast**	111.2 ± 5.4^ad^	146.7 ± 18.7^ac^	229.7 ± 18.7^b^	157.8 ± 13.2^cd^	74.0 ± 17.7^d^	16.45***
Mean Diurnal Range (°C)	**bio2**	0.99 ± 0.01^a^	1.17 ± 0.02^bc^	1.14 ± 0.02^b^	1.24 ± 0.02^c^	1.20 ± 0.03^bc^	45.11***
Isothermality (* 100)	**bio3**	37.3 ± 0.2^a^	40.2 ± 0.3^bc^	39.2 ± 0.3^b^	41.0 ± 0.3^c^	41.1 ± 0.5^c^	32.60***
Mean Temperature of Coldest Quarter (°C)	**bio11**	0.36 ± 0.02^a^	0.60 ± 0.05^b^	0.53 ± 0.02^b^	0.48 ± 0.03^ab^	0.64 ± 0.06^b^	11.02***
Precipitation of Driest Month (mm)	**bio14**	35.9 ± 1.4^a^	9.5 ± 1.7^b^	17.0 ± 2.2^bc^	21.5 ± 1.4^c^	19.4 ± 1.7^bc^	30.65***
Precipitation of Wettest Quarter (mm)	**bio16**	264.8 ± 5.2^a^	183.3 ± 11.7^b^	163.5 ± 8.0^b^	155.5 ± 5.8^b^	157.1 ± 6.1^b^	44.80***
Soil water capacity (v%)	**aw**	14.9 ± 0.1^a^	12.1 ± 0.2^b^	13.0 ± 0.3^b^	12.8 ± 0.2^b^	12.6 ± 0.3^b^	34.98***
Cation exchange capacity (cmolc/kg)	**cat**	20.8 ± 0.3^a^	19.5 ± 0.5^a^	16.8 ± 0.5^b^	17.4 ± 0.5^b^	17.9 ± 0.6^b^	18.11***
Soil pH (pH)	**ph**	6.7 ± 0.0^a^	7.3 ± 0.1^b^	7.4 ± 0.1^b^	7.6 ± 0.1^b^	7.4 ± 0.1^b^	25.75***
Clay content (w%)	**clay**	24.5 ± 0.3	25.6 ± 0.6	24.2 ± 0.7	24.2 ± 0.5	25.9 ± 0.5	1.21^n.s.^
Fragment content (v%)	**frag**	18.0 ± 0.4^ab^	18.6 ± 0.7^b^	15.6 ± 0.6^a^	17.4 ± 0.7^b^	18.6 ± 0.9^ab^	2.47*
Sand content (w%)	**sand**	37.9 ± 0.5	40.4 ± 0.8	40.8 ± 1.5	39.2 ± 0.9	36.6 ± 1.1	2.89^n.s.^
Soil texture (USDA system)	**text**	6.4 ± 0.1	6.2 ± 0.3	6.4 ± 0.3	7.0 ± 0.0	6.1 ± 0.4	1.91^n.s.^

The number of populations (N) and F value and significance levels (n.s. P > 0.05, * P < 0.05, *** P < 0.001) for the comparison among cytotypes are also provided. Different letters correspond to statistically significant differences.

Diploid individuals in Europe grow in habitats with significantly higher precipitation levels than the other cytotypes and in soils with the highest water retention and cation exchange values and the lowest pH levels (**Table 2**, Appendix 3). Diploids also grow in areas with significantly lower mean temperature in the coldest quarter, low-temperature diurnal range and lower isothermality than polyploids (**Table 2**, Appendix 3).

Environmental variables for polyploids largely overlap, although some trends are observed. Tetraploids tend to occur in habitats with higher values of precipitation in the wettest quarter, higher levels of cation exchange capacity and lower values of rainfall in the driest month than higher ploidy levels (**Table 2**, Appendix 3). The ecological attributes of the niche of hexaploids have similarities to all cytotypes, presenting high geographical segregation with the remaining cytotypes. Moreover, these populations had the highest distance to the coast (**Figure 1A**; **Table 2**, Appendix 3). Octoploids and decaploids occurred in a wide range of elevations. Their habitats were characterised by the highest values of isothermality and the lowest levels of precipitation for the wettest quarter. The distance to the coast of decaploids populations was the lowest among cytotypes (**Table 2**, Appendix 3).

### Ecological niche modelling

The predicted ecological niche of *L. suffruticosum s.l.* confirmed the distribution patterns in Europe, and the variables with the highest contribution to the model were isothermality, elevation and soil pH (**Figures 1A, C**; Appendix 4). The predicted suitable area was considerably larger than the area where the plant was found in North Africa (**Figuress 1A, C**).

Overall, each cytotype’s predicted distribution confirmed the parapatric distribution of *L. suffruticosum s.l.* cytotypes (**Figures 1A, D–H**). According to the models, suitable area for diploids could be found in the Iberian Peninsula, but mainly in the south of France, where diploid populations occupy the widest continuous area (**Figures 1A, D**). In North Africa and the Iberian Peninsula, diploids have a high probability of occurrence in mountainous regions (**Figure 1D**). The areas with the highest suitability of occurrence, were also those where most diploid populations were found (**Figures 1A, D**). The variables that mainly explained the predicted distribution of diploids were isothermality, precipitation in the wettest quarter, and soil pH (Appendix 4). The high contribution of soil pH is in line with the significantly lower soil pH observed in natural populations (**Figure 1D**; **Table 2**, Appendix 3).

For polyploids, the predicted suitable area for the Iberian Peninsula and North Africa suggested a high potential of expansion beyond their current range, as the areas with a high probability of occurrence were more extensive than the observed distributions (**Figures 1A, E–H**). Furthermore, models suggested the existence of suitable areas for octoploids and, to a less extent, decaploids in North Africa (**Figures 1A, G–H**). In tetraploids, the model was primarily explained by elevation and precipitation in the driest month, and in hexaploids, by elevation, precipitation in the wettest quarter and isothermality (Appendix 4). Octoploids were distributed primarily in arid inland regions. Still, the predicted suitability showed a larger area with a high probability of occurrence that expands to the coast and the north of the Iberian Peninsula. Also, in North Africa, where no octoploid populations were found, high suitability was detected in inner lands, especially in the eastern areas (**Figures 1A, G**). For decaploids, the model predicted suitable areas along the coast, which matches actual distribution in the Iberian Peninsula. Although no decaploid populations were found in North Africa, a few areas with high habitat suitability were also found along the coast (**Figures 1A, H**). In octoploids and decaploids, habitats were strongly influenced by temperature and water availability since variables related to precipitation and isothermality significantly contributed to the model’s prediction. Soil fragment content was also a relevant factor in predicting the niche of decaploids (Appendix 4).

### Niche equivalence and similarity tests

**Figure 2:**
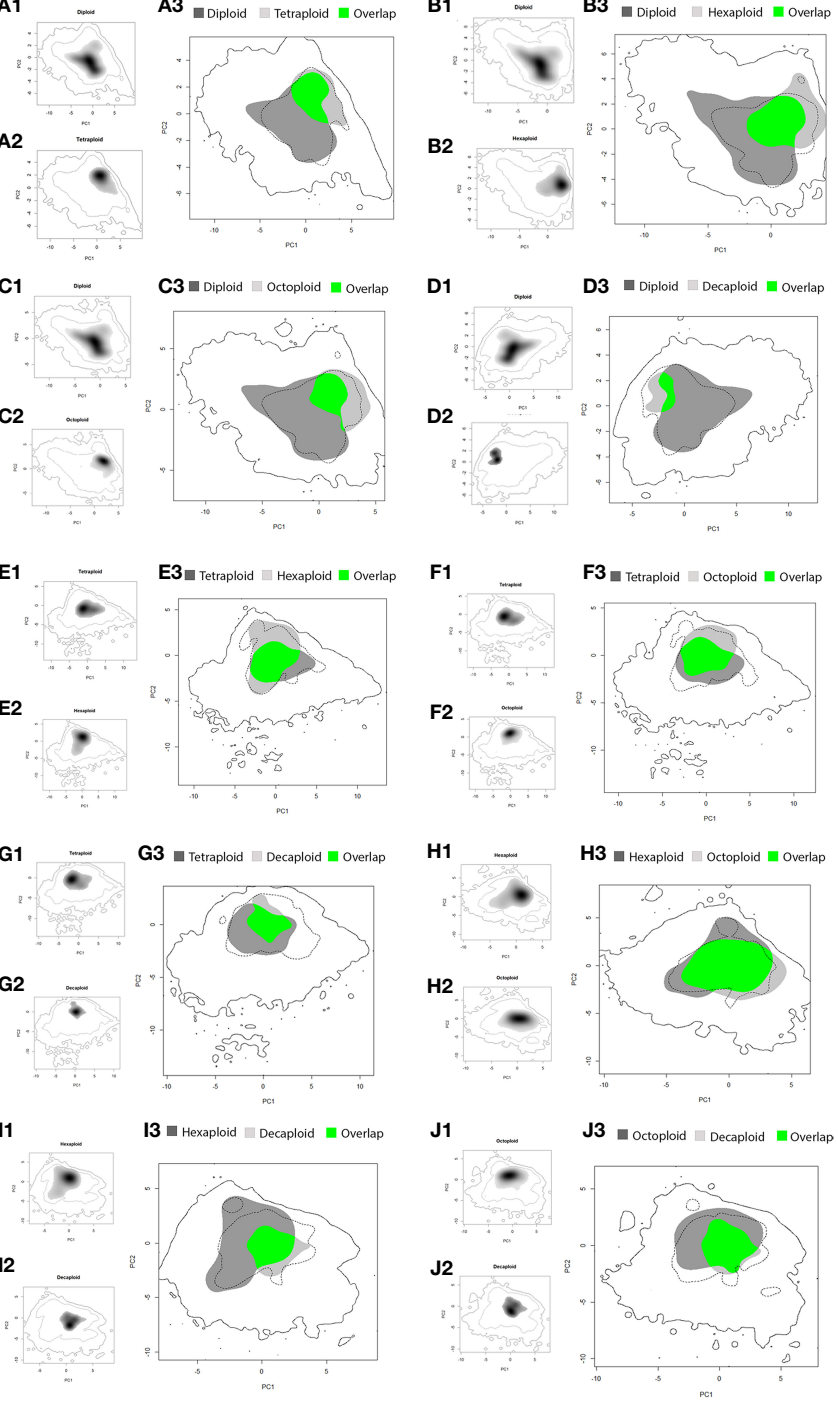
Comparison of ecological niche models for *Linum suffruticosum s.l.* pairs of cytotypes, based on the PCA of selected variables; diploid vs tetraploid **(A1-A3)**, diploid vs hexaploid **(B1-B3)**, diploid vs octoploid **(C1-C3)**, diploid vs decaploid **(D1-D3)**, tetraploid vs hexaploid **(E1-E3)**, tetraploid vs octoploid **(F1-F3)**, tetraploid vs decaploid **(G1-G3)**, hexaploid vs octoploid **(H1-H3)**, hexaploid vs decaploid **(I1-I3)**, octoploid vs decaploid **(J1-J3)**; coloured areas represent suitable habitats for cytotype 1 (light grey) and cytotype 2 (dark grey) and overlapping areas (green). The continuous line corresponds to the whole climatic space, while the dashed line indicates the 75^th^ percentile.

The amplitude of the niche of diploids was larger than that of the higher-ploidy cytotypes and presented low environmental niche overlap (diploid niche overlaps with other cytotype niches - 4*x*: 28.1%; 6*x*: 32.3%, 8*x*: 21.3%, 10*x*: 9.0%; **Table 3**; **Figure 2**). Thus, the ecological range of the polyploid’s niches was smaller than that of diploids (**Figures 2A1–D3**), and a high percentage of the polyploid’s environmental niches occurred within the diploid one (cytotype niche overlap with diploid niche - 4*x*: 58.9%; 6*x*: 60.1%; 8*x*: 51.4%; 10*x*: 51.7%; **Table 3**; **Figures 2A1–D3**). Comparing the diploid environmental niche with that of the polyploids demonstrated that the occurrence density in the ecological space was different, as showed by a low, but statistically significant, *D* value in the equivalence tests (**Table 3**; **Figures 2A1–A2; B1–B2; C1–C2; D1–D2**). Thus, polyploid niches occupy areas of the diploid niche with reduced density, corresponding to less optimal conditions for diploids (**Figure 2**). In addition, the first two axes of the PCA of the comparisons with the diploid niche explained a high percentage of the ecological variance (4*x*: 58.3%; 6*x*: 60.4%; 8*x*: 59.8%; 10*x*: 59.3%; **Table 3**; **Figures 2A1–D3**).

**Table 3:** **Equivalency (*D* and *P* values) and similarity (*P* value) tests for suitable habitat for each pair of cytotypes of *L. suffruticosum s.l.***.

A vs B	Equivalence test		Similarity test (*P* values)		% Niche overlap		% PCA	
	*D* value	*P* value	A–» B	B –» A	A–» B	B –» A	% PCA1	% PCA2
**2*x* vs 4*x***	**0.14**	**0.01**	0.82	0.81	28.1%	58.9%	35.8%	22.5%
**2*x* vs 6*x***	**0.11**	**0.01**	0.78	0.85	32.3%	60.1%	36.4%	24.0%
**2*x* vs 8*x***	**0.07**	**0.01**	0.82	0.75	21.3%	51.4%	37.0%	22.8%
**2*x* vs 10*x***	**0.02**	**0.01**	0.73	0.65	9.0%	51.7%	39.1%	20.2%
**4*x* vs 6*x***	**0.15**	**0.01**	0.82	0.79	66.7%	58.7%	30.5%	18.4%
**4*x* vs 8*x***	0.21	0.06	0.72	0.67	58.8%	68.7%	31.8%	17.5%
**4*x* vs 10*x***	**0.19**	**0.05**	0.88	0.85	35.8%	80.8%	33.8%	16.4%
**6*x* vs 8*x***	0.39	0.60	0.84	0.93	72.2%	79.3%	28.2%	21.8%
**6*x* vs 10*x***	**0.11**	**0.01**	0.80	0.77	26.6%	70.9%	27.7%	22.3%
**8*x* vs 10*x***	0.25	0.13	0.89	0.85	45.9%	89.3%	29.3%	21.8%

The percentage of niche overlap and variance explained by the first two axes of the Principal Component Analyses (PCA) are also presented. Abbreviations: 2x, diploids; 4x, tetraploids; 6x, hexaploids; 8x, octoploids; 10x, decaploids. Values with P < 0.05 are highlighted in bold.

The contribution of climatic and soil variables to the environmental space of tetraploids, hexaploids and octoploids differed: in the PCA1 axis, the octoploid’s environmental niche was different from that of the other cytotypes, and in the PCA2 axis, the hexaploid’s environmental niche was different from that of the other cytotypes. The environmental niche of decaploids was narrower than the other cytotypes for the PCA2 axis (**Figures 2D, G, I, J**). The environmental niche of tetraploids and hexaploids largely overlapped (66.7% and 58.7%, **Table 3**; **Figures 2E1–E3**), but the occurrences density in the ecological space of each cytotype was different, as demonstrated by the equivalency test (*D* = 0.15, *P <* 0.05, **Table 3**; **Figures 2E1, 2E2**). Comparing the environmental niches of tetraploids and octoploids revealed that the climatic niches are equivalent (*P >* 0.05, **Table 3**; **Figures 2E1–E3**). Indeed, the environmental niche of tetraploids and octoploids overlap (58.8% and 68.7%, **Table 3**; **Figure 2E3**). The environmental niches of tetraploids and decaploids are not equivalent (*D* = 0.19, *P <* 0.05, **Table 3**; **Figures 2F1–F3**). Even though the environmental niche of tetraploids had low overlap with that of decaploids (35.8%, **Table 3**), the niche of decaploids was within that of tetraploids (80.8%, **Table 3**; **Figure 2F3**). Hexaploids and octoploids have an environmental overlap (72.2% and 79.4%, **Table 3**), and their geographic niche is equivalent (*D* = 0.39, *P >* 0.05, **Table 3**), being also evident by similar occurrence densities (**Figures 2H1–H2**). By opposition, the geographic niche of hexaploids and decaploids was not equivalent, and their occurrences density was significantly different (*D* = 0.11, *P <* 0.05, **Table 3**; **Figures 2I1–I2**). The hexaploid niche has a low niche overlap within the decaploid niche (26.6%, **Table 3**; **Figure 2I3**); still, the ecological requirements of decaploids broadly fall within the niche of the hexaploids (70.9%, **Table 3**; **Figure 2I3**). Octoploids and decaploids presented geographic niche overlap, being equivalent (*D =* 0.25, *P >* 0.05). A high percentage of the environmental niche of decaploids is like the octoploids (89.3%), but only 45% of octoploids’ niche fall within the niche of the decaploid (**Table 3**; **Figures 2J1–J3**).

Although significant differences were observed in niche equivalency in some cytotype pairs, in niche similarity, the observed *D* values fall within the 95^th^ percentile of the simulated values, which indicates that cytotypes were not more similar (or different) from one another than expected after random sampling (**Table 3**).

## Discussion

This study revealed differences in the ecological attributes of *L. suffruticosum s.l.* cytotypes, with polyploids associated with habitats with increased drought (low precipitation and high temperatures), increased temperature ranges (both isothermality and mean diurnal temperature), higher soil pH, and decreased soil water and cation exchange capacities. These results could be explained as an adaptation of polyploids to dry and harsh environments. Despite the absence of environmental niche differences among most of the polyploids, the niche of the diploids differed significantly from that of the polyploids, being the widest among all cytotypes. Polyploids may have spread to environments less suitable for the diploids to escape inter-cytotypes competition. Additionally, in the two sides of the Mediterranean basin separated by the Strait of Gibraltar (SW Europe and NW Africa), the ecological niche of *L. suffruticosum s.l.* is different, as well as the niche of diploids and polyploids in each area. Below, we discuss the mechanisms underlying these results and their implications for understanding polyploid establishment and persistence.

### Ecological differences between diploids and polyploids

Recent detailed field surveys enabled to map *L. suffruticosum s.l.* cytotypes through its entire distribution range. The results here suggest that the parapatric distribution of cytotypes observed in the field (Afonso et al., 2021) can partly be explained by differences in the ecological niche. The habitats where diploids occur presented ecological dissimilarities compared to those where polyploids were found. Diploids were found in habitats with relative high precipitation, low temperatures and isothermality, higher soil water retention, and lower soil pH and cation exchange capacity than polyploids. By opposition, polyploids grow in drier and harsher habitats (low precipitation and high temperatures), with high isothermality, mean diurnal temperature, and soil pH. In fact, pH is a key predictor for the occurrence of many plant species since it affects nutrient availability (Wagner et al., 2017). In the Mediterranean region, the distribution patterns of other polyploid complexes have also been shown to be constrained by environmental variables related to precipitation and temperature imposed by the Mediterranean climate (Muñoz-Pajares et al., 2018; López-Jurado et al., 2019), given its high spatio-temporal dynamic nature (Nieto-Feliner, 2014; Cook et al., 2016). Also, it has already been shown that polyploids tend to grow in more specialised niches in more stressful habitats (Brochmann et al., 2004; Blaine Marchant et al., 2016; Hijmans et al., 2017).

Although both diploids and tetraploids grow in places with a mountain-influenced climate, diploids of *L. suffruticosum s.l.* were always found in populations at high elevations. Also, diploid plants are smaller than tetraploid ones (A. Afonso, personal observations). Many studies have demonstrated niche differentiation across altitudinal gradients, with diploids growing at high elevations and polyploids at lower elevations (Stebbins, 1971; Te Beest et al., 2012). Furthermore, we also observed that tetraploids are not as highly restricted in soil characteristics as diploids that only grow in habitats with the highest water retention and cation exchange values and low soil pH levels. This ability of tetraploids to colonise different soils could have allowed them to expand beyond the suitable areas of their diploid parentals and overcome the minority cytotype exclusion. Tetraploids, hexaploids, and decaploids occupy different geographic niches, suggesting a possible niche specialisation (Vamosi et al., 2014; Parisod and Broennimann, 2016). However, hexaploid and octoploid populations presented equivalent and similar environmental niches. The same was true for tetraploid and octoploid populations and octoploid and decaploid populations. The absence of environmental niche differentiation among polyploids was not completely unexpected, as the requirements of the higher-ploidy individuals might not differ from their lower-polyploid ancestors (Godsoe et al., 2013; Laport et al., 2016). As ecological preferences do not constitute a strong barrier in *L. suffruticosum s.l.* polyploids, the gene flow between individuals of neighbouring populations is still possible. Furthermore, it was shown that cytotypes are not reproductively isolated (Afonso, 2022).

Despite the differences in ecological requirements, diploids have a broader environmental niche breadth than polyploids, and polyploids occupy a part of the diploid’s niche. In practice, diploids and polyploids share the same environmental niche (niches were not more similar nor different than expected in a random sampling), and polyploids occur at marginal areas of the diploid niche, despite growing in other geographic areas (ecological niches were not equivalent). In young polyploid complexes (as *L. suffruticosum s.l.* seems to be, Ruiz-Martín et al., 2018; Maguilla et al., 2021), polyploids may partially occupy the niche of their progenitors, thus growing in climatic conditions of diploids as they did not have time yet to disperse further, specialise and/or completely diverge in their niche (Felber, 1991; Kim et al., 2012b; Glennon et al., 2014). Alternatively, polyploids could have diverged in their niche and later recolonised part of the diploid niche (Ståhlberg and Hedrén, 2009; Glennon et al., 2014).

Nevertheless, previous works have suggested that spatial segregation reflects ecological niche divergence and is one of the requirements for the successful establishment of polyploid lineages (Lumaret et al., 1987; Levin, 2002). Interestingly, as observed in *L. suffruticosum s.l.*, in other polyploid complexes, it was shown that the frequency of polyploid individuals increases at the periphery of parental ranges (Levin, 1975; Fowler and Levin, 1984; Felber, 1991), suggesting environmental specialisation (Knouft et al., 2012; Vamosi et al., 2014; Parisod and Broennimann, 2016). Several studies also indicated that spatial segregation could have resulted from the ability of polyploids to tolerate low nutrient levels, drought, and cold temperatures and colonise areas unfavourable or less favourable to their lower-ploidy progenitors (Levin, 2002; Maherali et al., 2009; Hao et al., 2013). Examples of environmental niche divergence between cytotypes have been reported in several polyploid complexes (Glennon et al., 2014; Thompson et al., 2014; Visger et al., 2016; Muñoz-Pajares et al., 2018), although it is difficult to separate the direct effects of WGD from subsequent evolutionary divergence (Maherali et al., 2009). The absence of niche specialisation of *L. suffruticosum s.l.* polyploids could be either because genome duplications did not generate significant direct physiological changes due to their recent origin (Ruiz-Martín et al., 2018; Maguilla et al., 2021) or because they might have been subjected to recurrent gene flow (Godsoe et al., 2013; Laport et al., 2016). The latter hypothesis is discussed below.

### Maintenance of the mosaic distribution of the polyploid complex

Previous field screenings of *L. suffruticosum s.l.* found that most populations were pure-ploidy populations. However, a few mixed-ploidy populations with minority cytotypes or aneuploids were also observed (Afonso et al., 2021). Despite being rare, the occurrence of mixed-ploidy populations (two diploid-tetraploid and one tetraploid-hexaploid) and minority cytotypes (namely, triploid, tetraploid, hexaploid, octoploid, and aneuploid individuals) can give us clues about how dynamic this polyploid complex can be. Despite the observed mosaic distribution, there is a clear contact zone between diploids and tetraploids in southern Spain (where these two cytotypes are abundant) and some contact areas in northern Spain (where diploids and tetraploids are scarce) and in North Africa (**Figure 1A**). The low number of diploid-tetraploid mixed populations (Afonso et al., 2021) suggests that the two cytotypes cannot occur in sympatry, likely because of the minority cytotype exclusion (Levin, 1975; Fowler and Levin, 1984; Husband, 2000; Levin, 2002). Although long-distance pollen flow and hybridisation between the two cytotypes cannot be excluded entirely, the presence of a few triploids in diploid populations suggests that tetraploids likely arose from the fusion of unreduced gametes, leading to a primary contact zone. Current diploid-tetraploid distribution could thus result from the combined effect of differences in environmental preferences and minority cytotype exclusion. However, other processes may further contribute to the distribution pattern of diploids and tetraploids, such as inter-cytotype competitive exclusion and/or divergent evolution.

Hexaploids occupy a large area, presenting the westernmost distribution in the Iberian Peninsula and being more geographically segregated from the others cytotypes in central Spain. There is a clear area of suitable habitats for hexaploids in central Spain, where most natural populations were found. Despite the occurrence of a tetraploid-hexaploid mixed population, their ecological niches were not equivalent. Similar results were found for hexaploid and decaploid niches. No hexaploid-decaploid mixed-ploidy population was found, as they occur far apart (Afonso et al., 2021). These observations suggest that hexaploids have suitable areas in regions not overlapping with the other cytotypes. Thus, they support the important role of environmental variables in defining their distribution. Octoploids and decaploids also have a clear area of suitable habitats, with high overlap between them (with 89.3% of the environmental niche of decaploids in the niche of octoploids and 45% of octoploid’s niche within the niche of the decaploid). Since there is no evidence of mixed-ploidy populations (Afonso et al., 2021), their distribution could be explained by the minority cytotype exclusion (Levin, 1975; Fowler and Levin, 1984; Husband, 2000; Levin, 2002). Different competitive abilities are expected to generate moving contact zones and expand the cytotype area until the environmental limit of the strongest competitor is reached (Maceira et al., 1993). However, considering the reproductive system of this species, with distyly being present in all populations and a strong self- and morph-incompatible system (Afonso, 2022), the absence of compatible mates might be critical during colonisation of new areas, contributing to more stable areas.

To sum up, for some cytotypes, there was a divergence of niche and colonisation of areas that were not favourable to the other cytotypes. Additionally, the existence of primarily pure populations in contact zones between cytotypes that show a large overlap of suitable niches supports the existence of minority cytotype exclusion. Nevertheless, the forces that maintain the dynamics of each contact zone will also depend on other factors, such as competition ability or reproductive strategies. Further investigation about the polyploids’ competitive abilities and reproductive strategies is needed in the future.

### Different environmental requirements across geographic areas – evolutionary implications

The distribution patterns of *L. suffruticosum s.l.* in the Iberian Peninsula and North Africa could be associated with different ecological preferences related to soil properties and climate. The morphological variability, geographical overlap, and high cytogenetic diversity detected in the field might indicate multiple origins of the polyploids from the same and/or from different progenitors (Nicholls, 1986; Ruiz-Martín, 2017; Afonso et al., 2021). The paleoclimatic history of the Mediterranean Basin includes important long-term changes, such as gradual global cooling and aridification (Zachos et al., 2008) and cyclical climatic changes (Jansson and Dynesius, 2002). The dynamic mosaic distribution of cytotypes could represent the result of different waves of colonisation and retractions following ice ages. The Mediterranean Basin has served as a refugium for many species during the Tertiary and the Quaternary, and it has been a reservoir for later colonisation during interglacial periods (Thompson, 2020). However, this complex is very recent, having probably originated at the beginning of the Pleistocene (Ruiz-Martín et al., 2018; Maguilla et al., 2021). The spread and diversification could be related to the dispersal through the strait of Gibraltar (open during the last ca. 5 Myr) and a fast adaptation to new environments. The recurrent and possible different origins of polyploids could explain the existence of both diploids and polyploids in both continents. As described above, changes in environmental requirements promoting eco-spatial segregation would increase the probability of the establishment and persistence of neopolyploids (Felber, 1991). Thus, ecological differentiation could have occurred not only among cytotypes between continents but also within continents since they might have different evolutionary histories and have been exposed to other selective pressures. An ongoing robust phylogeographic study is currently being developed and might, at least in part, address this question.

In W Europe, polyploids only occur in the Iberian Peninsula, while only diploids were detected in the rest of the European distribution. The potential niche of polyploids seems to restrict their distribution in Europe to the Southern side of the Pyrenees, suggesting that areas north and northeast of the Pyrenees are not suitable or are less suitable for polyploids (as also supported by the models presented here), or they had no time yet to colonize these areas. In other polyploid complexes where diploids grow in a higher elevation than polyploids, this distribution pattern suggests that diploids are old and probably well adapted to different areas over the entire distribution area (Theodoridis et al., 2013; Dai et al., 2020). However, as mentioned above, other factors could have been involved at contact areas. For example, the Pyrenees could have acted as a geographic barrier for polyploids to spread after their recent emergence in the Iberian Peninsula, leading to the sole existence of diploids beyond the mountain complex. Geographical barriers seem to have played a significant role in driving the emergence and establishment of polyploid complexes in the Mediterranean flora (Marques et al., 2018). Reciprocal transplant experiments are however needed to test this hypothesis.

The potential niche projected for polyploids in North Africa is much higher than what is observed in nature and in the records from the literature and online sources. Due to scarce information about the species occurrence in North Africa, the European populations were used to project the potential ecological niche in North Africa. As there might be differences in the origin and evolutionary history of the North African populations, the niche projection may not be as accurate as envisaged, and the results here should be considered with caution. The habitat suitability for *L. suffruticosum s.l.* in North Africa is much larger than what was sampled in the field for all cytotypes, with a high probability of habitat suitability even for cytotypes not reported for this area (*e.g.*, octoploid and decaploid). Fennane et al. (2007) reported the possible occurrence of the species further South in Morocco, but we were not able to find it during field sampling. Also, the sampled populations were very small, having a lower number of individuals than those usually found in the Iberian Peninsula (A. Afonso, personal observation). The climatic versus topographical heterogeneity in North Africa is much higher than in the Iberian Peninsula, which may be one of the reasons for the difficulty in correctly identifying the niche in this area. Furthermore, North African habitats are characterised by low precipitation, high minimum temperatures, and different soil attributes (higher soil pH, low cation exchange capability and water retention capacity, variable, and slightly higher classes of soil texture). Soil texture mainly influences the soil water capacity; therefore, it is an essential factor in the adaptation to Mediterranean dry biomes (Saxton and Rawls, 2006; Padilla and Pugnaire, 2007). Overall, these environmental variables might help explaining the high suitability of *L. suffruticosum s.l.* polyploids in this region.

In addition, overall, the species range appears to be limited by the presence of limestone and related substrates combined with the Mediterranean climate. Consequently, the species is scarce in other soil types, with populations almost absent in the western half of the Iberian Peninsula, where limestone areas are scarcer and restricted to some regions. Also, the species does not occur in the western Mediterranean islands (Balearic Islands, Corsica, Sardinia, Sicily), despite the availability of limestone soil and Mediterranean climate and the close connections between these islands and the continent during the Ice Ages due to lower sea level (Jansson and Dynesius, 2002; Zachos et al., 2008; Thompson, 2020). Once again, this might be related to the recent origin of the complex, i.e., recent studies showed that the complex might be originated in Pleistocene and could not had time to diversify (Maguilla et al., 2021). The geological and climatic context during the evolutionary history of *L. suffruticosum s.l.* and subsequent divergent evolution could have shaped its diversity. Biogeographical processes, including historical patterns of origin or migration, interactions among cytotypes, and divergence in levels of environmental tolerance, have been reported as the main factors determining the success of populations with different ploidies (Husband et al., 2013). Despite we suggest that various polyploidisation events have occurred in other geographical areas and biogeographical contexts, leading to differences in the predicted and observed niche of cytotypes in both sides of the Mediterranean Sea, molecular dating and biogeographical analyses along the distribution range of this complex are necessary to fully understand the evolutionary processes that have governed the current distribution patterns.

## Conclusions

This study revealed variations between diploid and polyploid ecological niches with differences in precipitation and temperature ranges. However, some higher-ploidy cytotypes had equivalent ecological niches but never co-occurred. In addition, differences among cytotypes of different geographical areas were found. Overall, these results support that particular ecological requirements played a role in the distribution of cytotypes, but the mosaic distribution could not be entirely explained based on environmental conditions. Together with ecological attributes, reproductive and competitive interactions among cytotypes could have shaped the current diversity and distribution patterns.

## Data availability statement

The original contributions presented in the study are included in the article/**Supplementary Material**. Further inquiries can be directed to the corresponding author.

## Author contributions

AA, SC, JL, and JA designed the experimental approach. AA, JL, AF, SL and JL conducted field collections. AA, MC and AF performed the niche modelling analyses. AA, with the collaboration of all co-authors, analyzed the data, discussed the results, and wrote the manuscript. All authors contributed to the article and approved the submitted version.
