# Erratum: Ecological niches in the polyploid complex *Linum suffruticosum s.l.*

**DOI:** 10.3389/fpls.2023.1249110

**Published:** 2023-07-10

**Authors:** Frontiers Production Office

**Affiliations:** Frontiers Media SA, Lausanne, Switzerland

**Keywords:** ecological niche, linum, Mediterranean region, niche modelling, polyploids

Due to a production error, the full funding statement was not provided. “This work is carried out at the R&D Unit Centre for Functional Ecology - Science for People and the Planet (CFE), with reference UIDB/04004/2020, financed by FCT/MCTES through national funds (PIDDAC)” and “by MINECO-MICINN-MICIU research grants CGL2013-45037-P, PGC2018 099608-B-100 and PID2021-122715NB-I00 to JA” were missed from the statement. The correct funding statement can be found below:

“This work is carried out at the R&D Unit Centre for Functional Ecology - Science for People and the Planet (CFE), with reference UIDB/04004/2020, financed by FCT/MCTES through national funds (PIDDAC). This research was financed by POPH/FSE funds by the Portuguese Foundation for Science and Technology with a doctoral grant to AA (SFRH/BD/108451/2015), by MINECO-MICINN-MICIU research grants CGL2013-45037-P, PGC2018 099608-B-100 and PID2021-122715NB-I00 to JA, and by Integrated Program of Scientific Research and Technological Development CULTIVAR (CENTRO-01-0145-FEDER-000020), co-financed by the Regional Operational Programme Centro 2020, Portugal 2020 and European Union, through European Fund for Regional Development (ERDF) to SC and MC.”

The publisher apologizes for this mistake.

