# Supplementary material for: Ecological niches in the polyploid complex Linum suffruticosum s.l
Source: Front Plant Sci. 2023 Apr 19;14:1148828. doi: 10.3389/fpls.2023.1148828 (PMC10154603; doi:10.3389/fpls.2023.1148828)
Supplement: Supplementary file 1 [file DataSheet_1.docx]

## Supplementary material

Appendix 1. Additional populations of *Linum suffruticosum s.l.* used in this study, including data from GBIF and populations sample in the field (field data) additionally to the ones available in Afonso *et al*. (2021). Information about the country, ploidy level, coordinates, and data source are provided. Abbreviations: 2*x*, diploid; - , no information available regarding ploidy level.

| **Country** | **Ploidy level** | **Coordinates (longitude, latitude)** | **Source** |
| --- | --- | --- | --- |
| Algeria | - | 35.00191,-1.69000 | GBIF |
| Algeria | - | 34.71121,-1.52919 | GBIF |
| Algeria | - | 32.47723,-0.88002 | GBIF |
| Algeria | - | 32.56142,-0.75479 | GBIF |
| Algeria | - | 34.67475,-0.62188 | GBIF |
| Algeria | - | 34.67438,-0.62158 | GBIF |
| Algeria | - | 35.29926,-0.55875 | GBIF |
| Algeria | - | 35.29924,-0.54844 | GBIF |
| Algeria | - | 33.55924,-0.31422 | GBIF |
| Algeria | - | 33.55934,-0.31375 | GBIF |
| Algeria | - | 33.55913,-0.31311 | GBIF |
| Algeria | - | 34.78790,-0.25771 | GBIF |
| Algeria | - | 35.15528,4.08760 | GBIF |
| France | - | 42.38316,0.79038 | field data |
| France | - | 42.14775,0.80809 | field data |
| France | - | 42.26159,1.55418 | field data |
| France | - | 42.2616,1.55418 | field data |
| France | - | 42.26391,1.5786 | field data |
| France | - | 42.34083,1.6773 | field data |
| France | - | 42.34104,1.71902 | field data |
| France | - | 42.12808,1.86343 | field data |
| France | - | 42.25406,1.87156 | field data |
| France | - | 41.88294,2.32505 | field data |
| France | - | 42.32940,2.46178 | field data |
| France | - | 44.26368,3.22603 | field data |
| France | - | 43.85580,3.40369 | field data |
| France | - | 43.85295,3.40748 | field data |
| France | - | 44.24528,5.13186 | field data |
| France | - | 43.81130,5.41738 | field data |
| France | - | 44.69188,5.67261 | field data |
| France | - | 43.33808,5.77677 | field data |
| France | - | 43.71561,6.48246 | field data |
| France | - | 43.95023,6.51151 | field data |
| France | - | 43.96913,6.77871 | field data |
| France | - | 43.81819,7.15852 | field data |
| France | - | 43.78156,7.25228 | field data |
| France | 2*x* | 45.34233,0.53009 | field data |
| France | 2*x* | 47.01359,0.54389 | field data |
| France | 2*x* | 44.72748,0.55015 | field data |
| France | 2*x* | 44.91382,0.91041 | field data |
| France | 2*x* | 44.57694,0.95173 | field data |
| France | 2*x* | 44.37685,0.99413 | field data |
| France | 2*x* | 45.08731,1.21225 | field data |
| France | 2*x* | 44.32375,1.45193 | field data |
| France | 2*x* | 42.26168,1.55408 | field data |
| France | 2*x* | 42.26406,1.57844 | field data |
| France | 2*x* | 42.25878,1.60694 | field data |
| France | 2*x* | 42.24306,1.65840 | field data |
| France | 2*x* | 42.35431,1.68983 | field data |
| France | 2*x* | 42.34280,1.71804 | field data |
| France | 2*x* | 44.00648,1.88058 | field data |
| France | 2*x* | 42.92768,2.22010 | field data |
| France | 2*x* | 44.35223,3.04405 | field data |
| France | 2*x* | 44.29072,3.11781 | field data |
| France | 2*x* | 44.31679,3.18623 | field data |
| France | 2*x* | 43.76217,3.19308 | field data |
| France | 2*x* | 43.89537,3.27788 | field data |
| France | 2*x* | 44.52045,3.30480 | field data |
| France | 2*x* | 44.53441,3.31566 | field data |
| France | 2*x* | 44.35740,3.39321 | field data |
| France | 2*x* | 43.85575,3.40342 | field data |
| France | 2*x* | 44.18306,3.42710 | field data |
| France | 2*x* | 43.79252,3.43457 | field data |
| France | 2*x* | 43.94450,3.75128 | field data |
| France | 2*x* | 43.81401,3.76189 | field data |
| France | 2*x* | 43.87777,3.79476 | field data |
| France | 2*x* | 43.73861,3.86829 | field data |
| France | 2*x* | 43.88470,3.87942 | field data |
| France | 2*x* | 44.24543,5.13175 | field data |
| France | 2*x* | 44.28870,5.15818 | field data |
| France | 2*x* | 44.02568,5.20353 | field data |
| France | 2*x* | 43.99158,5.24231 | field data |
| France | 2*x* | 43.99573,5.26753 | field data |
| France | 2*x* | 44.20839,5.30797 | field data |
| France | 2*x* | 43.91538,5.36244 | field data |
| France | 2*x* | 44.86936,5.55651 | field data |
| France | 2*x* | 44.75780,5.60397 | field data |
| France | 2*x* | 44.40102,5.60980 | field data |
| France | 2*x* | 43.49727,5.61766 | field data |
| France | 2*x* | 44.21340,5.82559 | field data |
| France | 2*x* | 44.42599,5.92640 | field data |
| France | 2*x* | 43.40018,5.95794 | field data |
| France | 2*x* | 44.56877,5.99975 | field data |
| France | 2*x* | 44.62321,6.05137 | field data |
| France | 2*x* | 43.77365,6.25733 | field data |
| France | 2*x* | 43.90471,6.26188 | field data |
| France | 2*x* | 44.57872,6.28205 | field data |
| France | 2*x* | 43.85326,6.29738 | field data |
| France | 2*x* | 43.83488,6.32799 | field data |
| France | 2*x* | 43.82121,6.33933 | field data |
| France | 2*x* | 43.59993,6.47773 | field data |
| France | 2*x* | 43.67915,6.80786 | field data |
| France | 2*x* | 43.94288,6.91903 | field data |
| France | 2*x* | 43.93502,6.94795 | field data |
| France | 2*x* | 43.75534,7.08371 | field data |
| France | 2*x* | 43.82441,7.14850 | field data |
| France | 2*x* | 43.78105,7.25274 | field data |
| France | 2*x* | 43.87132,7.39935 | field data |
| France | 2*x* | 43.77050,7.41429 | field data |
| Italy | - | 45.15230,7.05635 | field data |
| Italy | - | 44.20828,8.39238 | field data |
| Italy | - | 44.55103,8.77351 | field data |
| Italy | - | 44.51626,8.79681 | field data |
| Italy | 2*x* | 47.17707,0.83299 | field data |
| Italy | 2*x* | 46.98093,0.87023 | field data |
| Italy | 2*x* | 45.17736,0.89165 | field data |
| Italy | 2*x* | 44.14883,6.74478 | field data |
| Italy | 2*x* | 44.12930,6.91598 | field data |
| Italy | 2*x* | 44.08788,6.95363 | field data |
| Italy | 2*x* | 44.08885,7.03835 | field data |
| Italy | 2*x* | 44.35820,7.16899 | field data |
| Italy | 2*x* | 44.06465,7.25121 | field data |
| Italy | 2*x* | 44.93493,7.94928 | field data |
| Morocco | - | 30.62055,-9.36000 | field data |
| Morocco | - | 35.75000,-5.37000 | field data |
| Morocco | - | 32.60058,-4.81533 | field data |
| Morocco | - | 33.87000,-4.02949 | field data |
| Morocco | - | 34.81744,-2.41999 | field data |
| Spain | - | 36.51626,-6.13829 | field data |
| Spain | - | 36.84303,-4.81689 | field data |
| Spain | - | 36.84772,-4.80372 | field data |
| Spain | - | 38.61821,-4.11421 | field data |
| Spain | - | 36.83497,-3.96445 | field data |
| Spain | - | 36.84573,-3.71784 | field data |
| Spain | - | 37.67548,-3.63504 | field data |
| Spain | - | 37.08130,-3.53616 | field data |
| Spain | - | 38.35197,-3.52645 | field data |
| Spain | - | 37.04302,-3.52627 | field data |
| Spain | - | 37.07966,-3.50738 | field data |
| Spain | - | 37.13986,-3.48191 | field data |
| Spain | - | 37.07730,-3.48177 | field data |
| Spain | - | 37.11288,-3.45447 | field data |
| Spain | - | 40.28628,-3.45067 | field data |
| Spain | - | 37.31237,-3.40771 | field data |
| Spain | - | 40.40841,-3.28319 | field data |
| Spain | - | 42.71315,-3.28087 | field data |
| Spain | - | 42.30792,-3.26693 | field data |
| Spain | - | 40.28126,-3.22350 | field data |
| Spain | - | 41.25421,-3.21485 | field data |
| Spain | - | 41.29013,-3.12433 | field data |
| Spain | - | 38.88706,-3.05306 | field data |
| Spain | - | 41.23544,-3.04183 | field data |
| Spain | - | 37.39097,-2.99911 | field data |
| Spain | - | 37.27691,-2.97741 | field data |
| Spain | - | 39.51558,-2.88366 | field data |
| Spain | - | 37.44014,-2.88298 | field data |
| Spain | - | 38.01059,-2.86330 | field data |
| Spain | - | 40.28111,-2.84901 | field data |
| Spain | - | 37.05358,-2.82561 | field data |
| Spain | - | 37.03333,-2.80352 | field data |
| Spain | - | 37.02997,-2.77830 | field data |
| Spain | - | 42.68585,-2.62159 | field data |
| Spain | - | 36.93636,-2.60633 | field data |
| Spain | - | 40.52624,-2.52711 | field data |
| Spain | - | 40.63177,-2.50335 | field data |
| Spain | - | 42.64925,-2.47542 | field data |
| Spain | - | 42.61402,-2.42155 | field data |
| Spain | - | 37.79578,-2.28389 | field data |
| Spain | - | 40.60276,-2.16080 | field data |
| Spain | - | 37.70116,-2.14920 | field data |
| Spain | - | 39.86344,-2.13530 | field data |
| Spain | - | 38.03207,-1.73343 | field data |
| Spain | - | 41.86741,-1.60224 | field data |
| Spain | - | 40.84743,-1.46141 | field data |
| Spain | - | 41.18116,-1.45741 | field data |
| Spain | - | 39.58531,-1.15620 | field data |
| Spain | - | 39.10466,-1.03229 | field data |
| Spain | - | 40.84296,-1.02938 | field data |
| Spain | - | 42.49427,-0.80719 | field data |
| Spain | - | 40.82986,-0.79818 | field data |
| Spain | - | 42.38981,-0.71527 | field data |
| Spain | - | 42.50463,-0.63880 | field data |
| Spain | - | 42.53272,-0.54955 | field data |
| Spain | - | 39.71479,-0.53845 | field data |
| Spain | - | 38.64907,-0.41454 | field data |
| Spain | - | 39.88275,-0.37088 | field data |
| Spain | - | 41.98788,0.28381 | field data |
| Spain | - | 41.28413,0.52138 | field data |
| Spain | - | 41.83267,0.59080 | field data |
| Spain | - | 41.83264,0.59143 | field data |
| Spain | - | 42.46673,0.77404 | field data |

Appendix 2. Correlation matrix for the 19 bioclimatic variables extracted from the WorldClim database ([www.worldclim.org](http://www.worldclim.org)), and 18 topographic and soil conditions variables at two different depths (15 and 30 cm) from the World Soil Information ([www.isric.or](http://www.isric.or)) for *L. suffruticosum s.l.* occurrence data (Appendix 1). Variable codes are provided in the footnotes.

|  | **elevation** | **aspect** | **avg_ndvi_jn** | **aw_15cm** | **aw_30cm** | **bio1** | **bio10** | **bio11** | **bio12** |
| --- | --- | --- | --- | --- | --- | --- | --- | --- | --- |
| **aspect** | 0.002 |  |  |  |  |  |  |  |  |
| **avg_ndvi_jn** | 0.042 | -0.025 |  |  |  |  |  |  |  |
| **aw_15cm** | 0.184 | -0.004 | 0.630 |  |  |  |  |  |  |
| **aw_30cm** | 0.277 | -0.012 | 0.505 | 0.891 |  |  |  |  |  |
| **bio1** | -0.721 | 0.018 | -0.489 | -0.598 | -0.630 |  |  |  |  |
| **bio10** | -0.681 | 0.009 | -0.509 | -0.690 | -0.713 | 0.948 |  |  |  |
| **bio11** | -0.617 | 0.028 | -0.480 | -0.482 | -0.509 | 0.944 | 0.817 |  |  |
| **bio12** | 0.304 | 0.011 | 0.568 | 0.532 | 0.461 | -0.540 | -0.607 | -0.449 |  |
| **bio13** | 0.247 | 0.017 | 0.431 | 0.346 | 0.282 | -0.349 | -0.427 | -0.259 | 0.920 |
| **bio14** | 0.257 | -0.018 | 0.639 | 0.656 | 0.596 | -0.704 | -0.708 | -0.674 | 0.760 |
| **bio15** | -0.070 | 0.031 | -0.499 | -0.555 | -0.504 | 0.545 | 0.505 | 0.567 | -0.272 |
| **bio16** | 0.259 | 0.019 | 0.432 | 0.371 | 0.310 | -0.370 | -0.455 | -0.271 | 0.931 |
| **bio17** | 0.277 | -0.012 | 0.636 | 0.634 | 0.572 | -0.685 | -0.692 | -0.647 | 0.817 |
| **bio18** | 0.289 | -0.026 | 0.612 | 0.551 | 0.522 | -0.742 | -0.695 | -0.768 | 0.728 |
| **bio19** | 0.131 | 0.042 | 0.242 | 0.282 | 0.202 | -0.093 | -0.254 | 0.077 | 0.752 |
| **bio2** | -0.160 | 0.015 | -0.562 | -0.585 | -0.573 | 0.504 | 0.607 | 0.384 | -0.534 |
| **bio3** | -0.226 | 0.041 | -0.467 | -0.295 | -0.309 | 0.581 | 0.434 | 0.699 | -0.304 |
| **bio4** | 0.014 | -0.034 | 0.003 | -0.278 | -0.261 | -0.129 | 0.165 | -0.423 | -0.199 |
| **bio5** | -0.602 | 0.011 | -0.566 | -0.726 | -0.738 | 0.921 | 0.980 | 0.785 | -0.635 |
| **bio6** | -0.586 | 0.028 | -0.353 | -0.335 | -0.361 | 0.850 | 0.673 | 0.952 | -0.307 |
| **bio7** | -0.082 | -0.016 | -0.293 | -0.503 | -0.487 | 0.177 | 0.439 | -0.095 | -0.425 |
| **bio8** | -0.472 | -0.032 | 0.004 | -0.258 | -0.274 | 0.255 | 0.365 | 0.069 | -0.293 |
| **bio9** | -0.284 | 0.036 | -0.506 | -0.442 | -0.441 | 0.736 | 0.634 | 0.815 | -0.377 |
| **cfrag_15cm** | 0.592 | 0.015 | -0.129 | 0.028 | 0.081 | -0.212 | -0.257 | -0.071 | 0.141 |
| **cfrag_30cm** | 0.664 | 0.013 | -0.087 | 0.083 | 0.151 | -0.320 | -0.364 | -0.173 | 0.200 |
| **clay_30cm** | -0.459 | 0.007 | -0.291 | -0.518 | -0.600 | 0.646 | 0.679 | 0.556 | -0.340 |
| **clay15cm** | -0.366 | -0.009 | -0.164 | -0.402 | -0.440 | 0.429 | 0.493 | 0.320 | -0.282 |
| **depth_rock** | -0.135 | -0.021 | 0.305 | 0.225 | 0.205 | -0.230 | -0.173 | -0.314 | 0.121 |
| **dist_coast** | 0.073 | -0.007 | 0.175 | 0.232 | 0.218 | -0.415 | -0.303 | -0.502 | -0.007 |
| **ph_15cm** | -0.321 | -0.017 | -0.631 | -0.620 | -0.555 | 0.567 | 0.663 | 0.442 | -0.661 |
| **ph_30cm** | -0.304 | -0.020 | -0.635 | -0.619 | -0.540 | 0.545 | 0.645 | 0.417 | -0.664 |
| **sand_15cm** | 0.326 | 0.030 | -0.053 | 0.007 | 0.056 | -0.137 | -0.184 | -0.055 | 0.095 |
| **sand_30cm** | 0.407 | 0.024 | 0.014 | 0.084 | 0.154 | -0.267 | -0.305 | -0.184 | 0.147 |
| **slope** | 0.632 | -0.021 | 0.233 | 0.212 | 0.267 | -0.493 | -0.493 | -0.411 | 0.374 |
| **text_15cm** | 0.210 | 0.008 | 0.079 | 0.280 | 0.305 | -0.262 | -0.310 | -0.190 | 0.128 |
| **text_30cm** | 0.305 | -0.008 | 0.285 | 0.466 | 0.511 | -0.525 | -0.556 | -0.455 | 0.272 |
| **troca_cat15cm** | 0.373 | -0.026 | 0.172 | -0.059 | -0.003 | -0.342 | -0.264 | -0.377 | 0.226 |

Appendix 2. (Continuation)

|  | **bio13** | **bio14** | **bio15** | **bio16** | **bio17** | **bio18** | **bio19** | **bio2** | **bio3** |
| --- | --- | --- | --- | --- | --- | --- | --- | --- | --- |
| **aspect** |  |  |  |  |  |  |  |  |  |
| **avg_ndvi_jn** |  |  |  |  |  |  |  |  |  |
| **aw_15cm** |  |  |  |  |  |  |  |  |  |
| **aw_30cm** |  |  |  |  |  |  |  |  |  |
| **bio1** |  |  |  |  |  |  |  |  |  |
| **bio10** |  |  |  |  |  |  |  |  |  |
| **bio11** |  |  |  |  |  |  |  |  |  |
| **bio12** |  |  |  |  |  |  |  |  |  |
| **bio13** |  |  |  |  |  |  |  |  |  |
| **bio14** | 0.488 |  |  |  |  |  |  |  |  |
| **bio15** | 0.075 | -0.766 |  |  |  |  |  |  |  |
| **bio16** | 0.991 | 0.502 | 0.068 |  |  |  |  |  |  |
| **bio17** | 0.565 | 0.984 | -0.729 | 0.571 |  |  |  |  |  |
| **bio18** | 0.559 | 0.844 | -0.526 | 0.576 | 0.831 |  |  |  |  |
| **bio19** | 0.810 | 0.290 | 0.146 | 0.826 | 0.363 | 0.139 |  |  |  |
| **bio2** | -0.429 | -0.527 | 0.324 | -0.457 | -0.520 | -0.506 | -0.332 |  |  |
| **bio3** | -0.169 | -0.492 | 0.427 | -0.177 | -0.472 | -0.607 | 0.154 | 0.622 |  |
| **bio4** | -0.238 | 0.028 | -0.166 | -0.261 | 0.009 | 0.213 | -0.546 | 0.302 | -0.526 |
| **bio5** | -0.459 | -0.721 | 0.506 | -0.489 | -0.704 | -0.709 | -0.290 | 0.716 | 0.494 |
| **bio6** | -0.137 | -0.553 | 0.510 | -0.142 | -0.524 | -0.669 | 0.198 | 0.123 | 0.592 |
| **bio7** | -0.399 | -0.261 | 0.051 | -0.429 | -0.272 | -0.121 | -0.559 | 0.720 | -0.052 |
| **bio8** | -0.226 | -0.182 | 0.061 | -0.245 | -0.219 | 0.120 | -0.512 | 0.124 | -0.195 |
| **bio9** | -0.213 | -0.618 | 0.509 | -0.229 | -0.562 | -0.793 | 0.168 | 0.390 | 0.646 |
| **cfrag_15cm** | 0.197 | -0.063 | 0.251 | 0.208 | -0.029 | -0.025 | 0.227 | -0.011 | 0.150 |
| **cfrag_30cm** | 0.233 | 0.017 | 0.194 | 0.248 | 0.048 | 0.062 | 0.230 | -0.089 | 0.072 |
| **clay_30cm** | -0.250 | -0.360 | 0.228 | -0.280 | -0.338 | -0.453 | -0.130 | 0.357 | 0.238 |
| **clay15cm** | -0.267 | -0.167 | 0.017 | -0.292 | -0.164 | -0.254 | -0.241 | 0.223 | 0.025 |
| **depth_rock** | 0.004 | 0.329 | -0.429 | 0.002 | 0.303 | 0.336 | -0.161 | -0.165 | -0.313 |
| **dist_coast** | -0.159 | 0.299 | -0.387 | -0.132 | 0.225 | 0.365 | -0.243 | -0.104 | -0.404 |
| **ph_15cm** | -0.587 | -0.537 | 0.204 | -0.613 | -0.537 | -0.562 | -0.496 | 0.545 | 0.237 |
| **ph_30cm** | -0.595 | -0.527 | 0.194 | -0.620 | -0.530 | -0.547 | -0.513 | 0.543 | 0.221 |
| **sand_15cm** | 0.176 | -0.141 | 0.276 | 0.187 | -0.120 | -0.034 | 0.211 | 0.000 | 0.134 |
| **sand_30cm** | 0.196 | -0.041 | 0.188 | 0.210 | -0.027 | 0.075 | 0.184 | -0.077 | 0.036 |
| **slope** | 0.346 | 0.267 | -0.030 | 0.354 | 0.295 | 0.333 | 0.185 | -0.305 | -0.259 |
| **text_15cm** | 0.115 | 0.065 | -0.018 | 0.134 | 0.057 | 0.121 | 0.133 | -0.086 | 0.050 |
| **text_30cm** | 0.185 | 0.312 | -0.240 | 0.213 | 0.285 | 0.369 | 0.109 | -0.320 | -0.207 |
| **troca_cat15cm** | 0.184 | 0.276 | -0.117 | 0.172 | 0.286 | 0.327 | -0.051 | -0.226 | -0.413 |

Appendix 2. (Continuation)

|  | **bio4** | **bio5** | **bio6** | **bio7** | **bio8** | **bio9** | **cfrag_15cm** | **cfrag_30cm** | **clay_30cm** |
| --- | --- | --- | --- | --- | --- | --- | --- | --- | --- |
| **aspect** |  |  |  |  |  |  |  |  |  |
| **avg_ndvi_jn** |  |  |  |  |  |  |  |  |  |
| **aw_15cm** |  |  |  |  |  |  |  |  |  |
| **aw_30cm** |  |  |  |  |  |  |  |  |  |
| **bio1** |  |  |  |  |  |  |  |  |  |
| **bio10** |  |  |  |  |  |  |  |  |  |
| **bio11** |  |  |  |  |  |  |  |  |  |
| **bio12** |  |  |  |  |  |  |  |  |  |
| **bio13** |  |  |  |  |  |  |  |  |  |
| **bio14** |  |  |  |  |  |  |  |  |  |
| **bio15** |  |  |  |  |  |  |  |  |  |
| **bio16** |  |  |  |  |  |  |  |  |  |
| **bio17** |  |  |  |  |  |  |  |  |  |
| **bio18** |  |  |  |  |  |  |  |  |  |
| **bio19** |  |  |  |  |  |  |  |  |  |
| **bio2** |  |  |  |  |  |  |  |  |  |
| **bio3** |  |  |  |  |  |  |  |  |  |
| **bio4** |  |  |  |  |  |  |  |  |  |
| **bio5** | 0.201 |  |  |  |  |  |  |  |  |
| **bio6** | -0.578 | 0.614 |  |  |  |  |  |  |  |
| **bio7** | 0.864 | 0.521 | -0.344 |  |  |  |  |  |  |
| **bio8** | 0.447 | 0.329 | 0.005 | 0.386 |  |  |  |  |  |
| **bio9** | -0.391 | 0.638 | 0.770 | -0.072 | -0.270 |  |  |  |  |
| **cfrag_15cm** | -0.262 | -0.216 | -0.050 | -0.204 | -0.406 | 0.095 |  |  |  |
| **cfrag_30cm** | -0.254 | -0.321 | -0.132 | -0.240 | -0.420 | 0.011 | 0.981 |  |  |
| **clay_30cm** | 0.119 | 0.663 | 0.482 | 0.266 | 0.183 | 0.475 | -0.237 | -0.312 |  |
| **clay15cm** | 0.234 | 0.476 | 0.269 | 0.274 | 0.223 | 0.257 | -0.307 | -0.352 | 0.902 |
| **depth_rock** | 0.253 | -0.193 | -0.300 | 0.094 | 0.237 | -0.409 | -0.377 | -0.352 | -0.083 |
| **dist_coast** | 0.376 | -0.305 | -0.541 | 0.222 | 0.181 | -0.533 | -0.090 | -0.064 | -0.331 |
| **ph_15cm** | 0.301 | 0.694 | 0.305 | 0.497 | 0.267 | 0.411 | -0.240 | -0.292 | 0.638 |
| **ph_30cm** | 0.316 | 0.679 | 0.279 | 0.507 | 0.271 | 0.390 | -0.242 | -0.289 | 0.613 |
| **sand_15cm** | -0.190 | -0.155 | -0.050 | -0.127 | -0.165 | 0.033 | 0.300 | 0.319 | -0.604 |
| **sand_30cm** | -0.158 | -0.274 | -0.164 | -0.147 | -0.170 | -0.078 | 0.303 | 0.340 | -0.698 |
| **slope** | -0.054 | -0.473 | -0.327 | -0.210 | -0.262 | -0.236 | 0.513 | 0.574 | -0.287 |
| **text_15cm** | -0.169 | -0.290 | -0.178 | -0.152 | -0.136 | -0.170 | 0.188 | 0.208 | -0.660 |
| **text_30cm** | -0.102 | -0.553 | -0.392 | -0.234 | -0.132 | -0.414 | 0.116 | 0.172 | -0.790 |
| **troca_cat15cm** | 0.251 | -0.258 | -0.323 | 0.041 | -0.004 | -0.264 | 0.109 | 0.169 | 0.196 |

Appendix 2. (Continuation)

|  | **Clay 15cm** | **Depth _rock** | **Dist _coast** | **Ph**  **_15cm** | **Ph**  **_30cm** | **Sand**  **_15cm** | **Sand**  **_30cm** | **slope** | **Text**  **_15cm** | **Text**  **_30cm** |
| --- | --- | --- | --- | --- | --- | --- | --- | --- | --- | --- |
| **aspect** |  |  |  |  |  |  |  |  |  |  |
| **avg_ndvi_jn** |  |  |  |  |  |  |  |  |  |  |
| **aw_15cm** |  |  |  |  |  |  |  |  |  |  |
| **aw_30cm** |  |  |  |  |  |  |  |  |  |  |
| **bio1** |  |  |  |  |  |  |  |  |  |  |
| **bio10** |  |  |  |  |  |  |  |  |  |  |
| **bio11** |  |  |  |  |  |  |  |  |  |  |
| **bio12** |  |  |  |  |  |  |  |  |  |  |
| **bio13** |  |  |  |  |  |  |  |  |  |  |
| **bio14** |  |  |  |  |  |  |  |  |  |  |
| **bio15** |  |  |  |  |  |  |  |  |  |  |
| **bio16** |  |  |  |  |  |  |  |  |  |  |
| **bio17** |  |  |  |  |  |  |  |  |  |  |
| **bio18** |  |  |  |  |  |  |  |  |  |  |
| **bio19** |  |  |  |  |  |  |  |  |  |  |
| **bio2** |  |  |  |  |  |  |  |  |  |  |
| **bio3** |  |  |  |  |  |  |  |  |  |  |
| **bio4** |  |  |  |  |  |  |  |  |  |  |
| **bio5** |  |  |  |  |  |  |  |  |  |  |
| **bio6** |  |  |  |  |  |  |  |  |  |  |
| **bio7** |  |  |  |  |  |  |  |  |  |  |
| **bio8** |  |  |  |  |  |  |  |  |  |  |
| **bio9** |  |  |  |  |  |  |  |  |  |  |
| **cfrag_15cm** |  |  |  |  |  |  |  |  |  |  |
| **cfrag_30cm** |  |  |  |  |  |  |  |  |  |  |
| **clay_30cm** |  |  |  |  |  |  |  |  |  |  |
| **clay15cm** |  |  |  |  |  |  |  |  |  |  |
| **depth_rock** | 0.103 |  |  |  |  |  |  |  |  |  |
| **dist_coast** | -0.200 | 0.201 |  |  |  |  |  |  |  |  |
| **ph_15cm** | 0.633 | 0.026 | -0.181 |  |  |  |  |  |  |  |
| **ph_30cm** | 0.618 | 0.035 | -0.161 | 0.994 |  |  |  |  |  |  |
| **sand_15cm** | -0.765 | -0.250 | -0.001 | -0.400 | -0.387 |  |  |  |  |  |
| **sand_30cm** | -0.788 | -0.188 | 0.063 | -0.450 | -0.432 | 0.982 |  |  |  |  |
| **slope** | -0.215 | -0.089 | -0.082 | -0.336 | -0.332 | 0.191 | 0.252 |  |  |  |
| **text_15cm** | -0.751 | -0.051 | 0.182 | -0.417 | -0.408 | 0.578 | 0.581 | 0.072 |  |  |
| **text_30cm** | -0.705 | 0.149 | 0.308 | -0.517 | -0.499 | 0.430 | 0.505 | 0.161 | 0.637 |  |
| **troca_cat15cm** | 0.392 | 0.148 | -0.065 | 0.082 | 0.090 | -0.301 | -0.225 | 0.443 | -0.402 | -0.212 |

Elevation, ele; exposition, aspect; average Normalized Difference Vegetation Index of the last 10 days of June of the last 5 years, avg_ndvi_jn; soil water capacity at 15 cm, aw_15cm; soil water capacity at 30 cm, aw_30cm; Annual Mean Temperature, bio1; Mean Temperature of Warmest Quarter, bio10; Mean Temperature of Coldest Quarter, bio11; Annual Precipitation, bio12; Precipitation of Wettest Month, bio13; Precipitation of Driest Month, bio14; Precipitation Seasonality, bio15; Precipitation of Wettest Quarter, bio16; Precipitation of Driest Quarter, bio17; Precipitation of Warmest Quarter, bio18; Precipitation of Coldest Quarter, bio19; Mean Diurnal Range, bio2; Isothermality, bio3; Temperature Seasonality, bio4; Max Temperature of Warmest Month, bio5; Min Temperature of Coldest Month, bio6; Temperature Annual Range, bio7; Mean Temperature of Wettest Quarter, bio8; Mean Temperature of Driest Quarter, bio9; Fragment content at 15 cm, cfrag_15cm; Fragment content at 30 cm, cfrag_30cm; Clay content at 15 cm, clay_15cm; Clay content at 30 cm, clay_30cm; Absolut depth to rock, depth_rock; distance to the coast, dist_coast; Soil pH at 15 cm, ph_15cm; Soil pH at 30 cm, ph_30cm; Sand content at 15 cm, sand_15cm; Sand content at 30 cm, sand_30cm; slope, slope; Soil texture at 15 cm, text_15cm; Soil texture at 30 cm, text_30cm; Cation exchange capacity at 15 cm, troca_cat15cm.

**Appendix 3.** Minimum (min) and maximum (max) values of selected variables used to characterise the niche of *Linum suffruticosum s.l.* cytotypes.

| Variables | CODE | Diploids  (min-max) | Tetraploids  (min-max) | Hexaploids  (min-max) | Octoploids  (min-max) | Decaploids  (min-max) |
| --- | --- | --- | --- | --- | --- | --- |
| Elevation (metres) - ele | ele | 52.0-2599.0 | 46.0-1738.0 | 438.0-1151.0 | 367.001315.0 | 32.0-966.0 |
| Distance to the coast (km) - dcoast | dcoast | 3.0-307.1 | 3.13-306.3 | 27.4-379.4 | 61.7-293.5 | 3.0-221.5 |
| Mean Diurnal Range (°C) – bio2 | bio2 | 0.6-1.3 | 0.8-1.3 | 0.9-1.3 | 0.9-1.4 | 0.6-1.4 |
| Isothermality (* 100) – bio3 | bio3 | 28.0-43.0 | 0.0-44.0 | 36.0-43.0 | 38.0-44.0 | 28.0-43.0 |
| Mean Temperature of Coldest Quarter (°C) – bio11 | bio11 | -0.5-1.0 | 0.2-1.2 | 0.3-1.1 | 0.3-0.9 | 0.4-1.0 |
| Precipitation of Driest Month (mm) – bio 14 | bio14 | 2.0-69.0 | 1.0-33.0 | 2.0-44.0 | 10.0-41.0 | 1.0-32.0 |
| Precipitation of Wettest Quarter (mm) – bio 16 | bio16 | 118.0-485.0 | 73.0-344.0 | 115.0-271.0 | 122.0-249.0 | 121.0-198.0 |
| Soil water capacity (v%) - aw | aw | 11.0-18.0 | 10.0-14.0 | 11.0-16.0 | 12.0-16.0 | 10.0-14.0 |
| Cation exchange capacity (cmolc/kg) - cat | cat | 13.0-30.0 | 14.0-25.0 | 13.0-21.0 | 14.0-23.0 | 14.0-22.0 |
| Soil pH (pH) - ph | ph | 5.5-8.0 | 6.3-8.0 | 5.7-8.1 | 5.7-8.1 | 5.7-7.9 |
| Clay content (w%) - clay | clay | 13.0-34.0 | 20.0-34.0 | 17.0-32.0 | 20.0-29.0 | 14.0-28.0 |
| Fragment content (v%) - frag | frag | 8.0-28.0 | 14.0-26.0 | 11.0-21.0 | 13.0-25.0 | 10.0-24.0 |
| Sand content (w%) - sand | sand | 25.0-52.0 | 27.0-47.0 | 30.0-59.0 | 28.0-46.0 | 23.0-43.0 |
| Soil texture (USDA system) - text | text | 4.0-8.0 | 4.0-7.0 | 4.0-9.0 | 7.0-7.0 | 4.0-7.0 |

Appendix 4. Contribution of variables in the models of *Linum suffruticosum s.l.* and of each cytotype. In bold are highlighted the variables with the highest contribution in each model. Abbreviations: 2*x*, diploids; 4*x*, tetraploids; 6*x*, hexaploids; 8*x*, octoploids; 10*x*, decaploids.

| **Variables** | **CODE** | ***L. suffruticosum* *s.l.*** | **2*x*** | **4*x*** | **6*x*** | **8*x*** | **10*x*** |
| --- | --- | --- | --- | --- | --- | --- | --- |
| Elevation (metres) | ele | **0.240** | 0.172 | **0.706** | **0.396** | 0.141 | 0.074 |
| Distance to the coast (km) | dcoast | 0.062 | 0.125 | 0.169 | 0.031 | **0.223** | **0.464** |
| Mean Diurnal Range (°C) | bio2 | 0.006 | 0.088 | **0.256** | 0.047 | 0.087 | 0.000 |
| Isothermality (* 100) | bio3 | **0.312** | **0.469** | 0.109 | **0.212** | **0.435** | **0.530** |
| Mean Temperature of Coldest Quarter (°C) | bio11 | 0.159 | 0.189 | 0.000 | 0.092 | 0.000 | 0.158 |
| Precipitation of Driest Month (mm) | bio14 | 0.032 | 0.055 | **0.717** | 0.082 | **0.444** | 0.114 |
| Precipitation of Wettest Quarter (mm) | bio16 | 0.047 | **0.206** | 0.012 | **0.250** | 0.173 | **0.584** |
| Soil water capacity (v%) | aw | 0.033 | 0.182 | 0.101 | 0.001 | **0.225** | 0.192 |
| Cation exchange capacity (cmolc/kg) | cat | 0.182 | 0.155 | **0.310** | 0.076 | 0.001 | 0.045 |
| Soil pH (pH) | ph | **0.203** | **0.345** | 0.005 | 0.042 | 0.095 | 0.000 |
| Clay content (w%) | clay | 0.014 | 0.063 | 0.000 | 0.000 | 0.000 | 0.000 |
| Fragment content (v%) | frag | 0.100 | 0.038 | 0.000 | 0.054 | 0.043 | **0.528** |
| Sand content (w%) | sand | 0.068 | 0.116 | 0.002 | 0.000 | 0.012 | 0.122 |
| Soil texture (USDA system) | text | 0.001 | 0.007 | 0.083 | 0.036 | 0.002 | 0.001 |
